# Precision of tissue patterning is controlled by dynamical properties of gene regulatory networks

**DOI:** 10.1242/dev.197566

**Published:** 2021-02-25

**Authors:** Katherine Exelby, Edgar Herrera-Delgado, Lorena Garcia Perez, Ruben Perez-Carrasco, Andreas Sagner, Vicki Metzis, Peter Sollich, James Briscoe

**Affiliations:** 1 Developmental Dynamics Laboratory, The Francis Crick Institute, 1 Midland Road, London NW1 1AT, UK; 2 Department of Mathematics, King's College London, Strand, London WC2R 2LS, UK; 3 Genetics and Developmental Biology Unit, Institut Curie, 26 Rue d'Ulm, Paris 75005, France; 4 Department of Life Sciences, Imperial College London, London SW7 2BU, UK; 5 School of Medical Sciences, Faculty of Biology, Medicine and Health, University of Manchester, Manchester M13 9PL, United Kingdom; 6 Faculty of Medicine, Institute of Clinical Sciences, Institute of Clinical Sciences, Imperial College London, London W12 0NN, UK; 7 Faculty of Physics, Institute for Theoretical Physics, Georg-August-University Göttingen, Friedrich-Hund-Platz 1, 37077 Göttingen, Germany

**Keywords:** cis regulatory elements, Dynamical systems theory, Gene regulatory network, Morphogen signaling, Neural tube

## Abstract

During development, gene regulatory networks allocate cell fates by partitioning tissues into spatially organised domains of gene expression. How the sharp boundaries that delineate these gene expression patterns arise, despite the stochasticity associated with gene regulation, is poorly understood. We show, in the vertebrate neural tube, using perturbations of coding and regulatory regions, that the structure of the regulatory network contributes to boundary precision. This is achieved, not by reducing noise in individual genes, but by the configuration of the network modulating the ability of stochastic fluctuations to initiate gene expression changes. We use a computational screen to identify network properties that influence boundary precision, revealing two dynamical mechanisms by which small gene circuits attenuate the effect of noise in order to increase patterning precision. These results highlight design principles of gene regulatory networks that produce precise patterns of gene expression.

## Abstract (precis)

**Summary:** Experiments and modeling reveal sharp boundaries of gene expression in the vertebrate neural tube depend on the dynamics of the gene regulatory network that patterns the tissue.

## INTRODUCTION

Embryos are characterised by remarkably organised and reproducible patterns of cellular differentiation. An illustration of this accuracy are the sharp boundaries of gene expression observed in many developing tissues. These patterns are determined by gene regulatory networks (GRNs), governed by secreted developmental signals (Davidson, 2010), raising the question of how precision is achieved in spite of the biological noise and inherent stochastic fluctuations associated with the regulation of gene expression (Raser and O'Shea, 2005).

A popular metaphor for the process of developmental pattern formation is the Waddington landscape, in which the differentiation trajectory of a cell is conceived as a ball rolling down a landscape of bifurcating valleys (Waddington, 1957). In this representation, the landscape is shaped by the GRN with the valleys representing cell fates and developmental signals allocating cell identity by determining the valley a cell enters. This can be formalised more rigorously by describing the GRN using dynamical systems theory such that Waddingtonian valleys correspond to the attractor states of the GRN (Enver et al., 2009; Wang et al., 2011; Balázsi et al., 2011; Zhou et al., 2012). In this view, cells can be driven out of a valley into an adjacent attractor, thus producing a change in identity, not only by developmental signals but also by gene expression noise.

How is noise buffered in developing tissues to ensure that developmental signals generate precise and reproducible patterns of gene expression? For individual genes, the activity of redundant regulatory elements (so-called shadow enhancers), the three-dimensional architecture of the genome, the presence of multiple alleles, and the effect of RNA processing, have all been proposed to buffer fluctuations and increase the robustness of gene expression (Perry et al., 2010; Frankel et al., 2010; Lagha et al., 2012; Little et al., 2013; Battich et al., 2015; Cannavò et al., 2016; Dickel et al., 2018; Osterwalder et al., 2018; Paliou et al., 2019; Tsai et al., 2019). At the level of the tissue, mechanisms that regulate the shape, steepness or variance of gradients have been explored and their effect on the precision of gene expression detailed (Bollenbach et al., 2008; Sokolowski et al., 2012; Tkačik et al., 2015; Zagorski et al., 2017; Lucas et al., 2018). Several mechanisms, including differential adhesion, mechanical barriers and juxtacrine signalling, have been proposed to correct anomalies and enhance precision, once cellular patterning has been initiated (Xu et al., 1999; Standley et al., 2001; Rudolf et al., 2015; Dahmann et al., 2011; Addison et al., 2018). In addition, theoretical studies have suggested that the structure and activity of GRNs can also affect precision (Chalancon et al., 2012; Lo et al., 2015; Perez-Carrasco et al., 2016). However, experimental evidence to support this remains elusive.

**Fig. 1.**
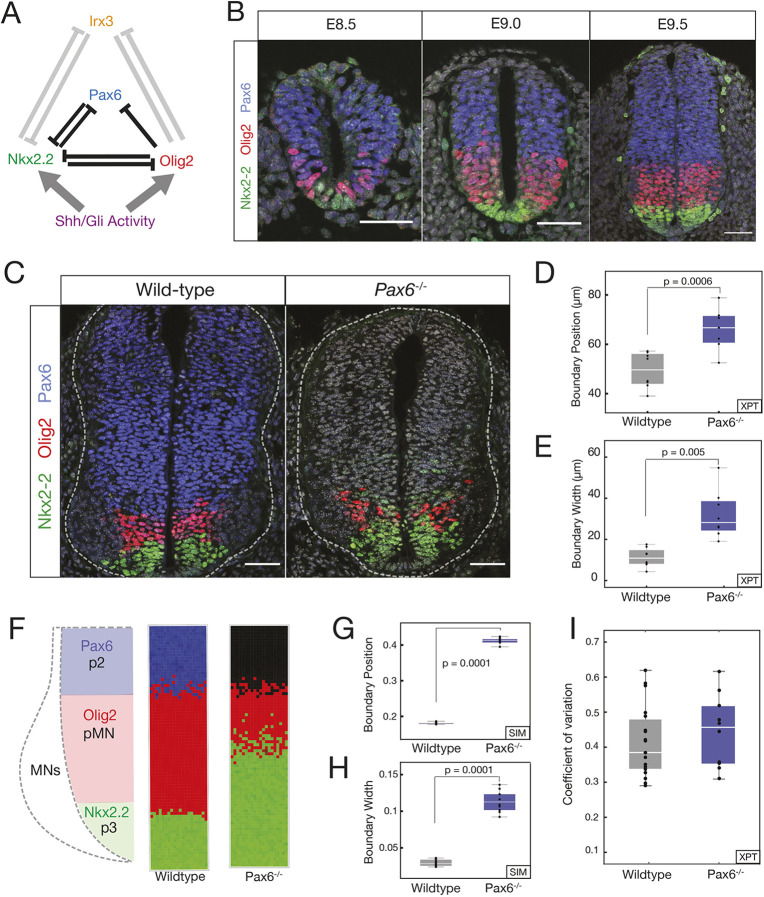
**Pax6 contributes to boundary precision.** (A) Schematic of the GRN responsible for positioning the p3 and pMN domains. (B) Immunofluorescence assays of Pax6 (blue), Olig2 (red) and Nkx2.2 (green) in neural progenitors from E8.5 to E9.5. (C) WT and Pax6^−/−^ embryos assayed for Olig2, Pax6 and Nkx2.2. (D) Position of the pMN-p3 boundary in WT (grey) and Pax6^−/−^ (blue). *n*=7 (WT), *n*=8 (Pax6^−/−^), *P*=0.005. (E) Width of pMN-p3 boundary in WT (grey) and Pax6^−/−^ (blue) (*P*=0.0006). (F) Stochastic simulations of the GRN in WT (middle) and Pax6^−/−^ (right). (G,H) Boundary position and width from simulations. Width is given as the fraction of total neural tube size. *n*=10 (WT), *n*=10 (Pax6^−/−^), *P*=0.0001 for position and boundary width. (I) CV of Olig2 levels for WT and Pax6^−/−^ (*P*=0.422). Box plots show upper and lower quartile and mean. Statistical significance was determined using a Mann–Whitney test. Scale bars: 50 μm

The developing vertebrate neural tube offers the opportunity to test the role of GRNs in the precision of patterning. The neural tube GRN partitions neural progenitors into discrete domains of gene expression arrayed along the dorsal-ventral axis (Sagner and Briscoe, 2019). The boundaries between these domains are clearly delineated and accurately positioned (Kicheva et al., 2014), resulting in sharp spatial transitions in gene expression that produce characteristic stripes of molecularly distinct cells. In the ventral neural tube, the secreted ligand Sonic Hedgehog (Shh), emanating from the notochord and floor plate, located at the ventral pole, controls the pattern forming GRN (Fig. 1A). The regulatory interactions between the transcription factors (TFs) comprising the GRN explain the dynamics of gene expression in the ventral neural tube and produce the genetic toggle switches that result in discrete transitions in gene expression in individual cells (Balaskas et al., 2012). The network includes the TFs Pax6, Olig2, Irx3 and Nkx2.2. Irx3 represses Olig2 Novitch et al. (2001), and Nkx2.2 is repressed by Pax6, Olig2 and Irx3 (Briscoe et al., 1999, 2000; Novitch et al., 2001; Balaskas et al., 2012). In the absence of Shh signaling, progenitors express Pax6 and Irx3. Moderate levels of Shh signalling are sufficient to induce Olig2 expression and repress Irx3 to specify motor neuron progenitors (pMN) (Ericson et al., 1997; Briscoe et al., 2000; Novitch et al., 2001; Balaskas et al., 2012). In response to high and sustained levels of Shh signalling, Nkx2.2 is induced and inhibits the expression of Pax6 and Olig2, which then generates p3 progenitors and delineates the p3-pMN boundary (Fig. 1B). In embryos lacking Pax6, the domain of Nkx2.2 expression expands, resulting in a decrease in Olig2 expression and a dorsal shift in the p3-pMN boundary (Ericson et al., 1997; Novitch et al., 2001; Balaskas et al., 2012).

In addition to the change in the position of the p3-pMN boundary, the loss of Pax6 also results in decreased precision of the p3-pMN boundary, with noticeably more intermixing of cells (Ericson et al., 1997; Briscoe et al., 2000; Novitch et al., 2001; Balaskas et al., 2012). Here, we set out to understand how this loss of precision occurs. We hypothesised that stochastic fluctuations in gene expression, coupled with changes in the dynamics of the GRN in the absence of Pax6, account for the decreased boundary precision. We provide a combination of experiments, data analysis and theory that are consistent with this idea. We also found that perturbing the regulatory input onto Olig2, by deleting a single cis-regulatory element, altered the dynamics of the GRN and decreased the precision of the p3-pMN boundary. The decreased precision was not a result of increased noise in the expression of individual genes. Instead, the absence of the Olig2 regulatory element, similar to the loss of Pax6, changed the overall configuration of the stochastic fluctuations by altering the relative size of fluctuations of the genes, which resulted in a change in the direction of stochastic fluctuations in gene expression space and made transitions from a pMN to p3 state more likely. A computational screen for networks that generate precise boundaries supported this idea and revealed two dynamical mechanisms by which small gene circuits attenuate the effect of noise in order to increase patterning precision. Thus, although mechanisms necessitating additional signals, differential adhesion or cell mechanics are often invoked to explain the precision of tissue patterning, our analysis suggests that the intrinsic properties of a GRN can also enhance boundary precision.

## RESULTS

### Pax6 contributes to p3-pMN boundary precision

We assayed the precision of the boundary between p3 (Nkx2.2 expressing) and pMN (Olig2 and Pax6 expressing) in the ventral neural tube. Consistent with previous reports (Ericson et al., 1997; Balaskas et al., 2012), compared with wild-type (WT) mouse embryos, the precision of the boundary between p3-pMN domains was decreased in embryos lacking Pax6, resulting in more intermixing of cells expressing Olig2 or Nkx2.2 (Fig. 1C) (Ericson et al., 1997; Briscoe et al., 2000; Balaskas et al., 2012). Measurements of the dorsal-ventral width of the region that contains both Nkx2.2- and Olig2-expressing cells in WT and Pax6 mutant embryos (Materials and Methods) indicated that between embryonic day (E)9.0 (9 days after fertilisation) and E10.5, the width of the pMN-p3 boundary region is wider in Pax6^−/−^ embryos, consistent with a loss of precision (Fig. 1D,E; Fig. S1).

We hypothesised that the decreased precision of the Nkx2.2 boundary, leading to the increased width in Pax6^−/−^ embryos (Fig. 1C), could be explained by noise in gene expression in the GRN. Previously, we established a deterministic model of the GRN, based on coupled ordinary differential equations (ODEs), that replicated the response of the network to Shh signalling and the shifts in boundary position in mutant embryos, including Pax6^−/−^ (Panovska-Griffiths et al., 2013; Balaskas et al., 2012; Cohen et al., 2014). This model recapitulated the pMN and p3 steady states. The analysis also produced a region of bistability in which both the pMN and p3 states were stable; however, owing to the initial conditions and deterministic behaviour, the system always adopted a pMN state in the bistable region. We reasoned that fluctuations in gene expression could result in noise driven transitions within the bistable region from a pMN state to a p3 identity. (For a glossary of dynamical systems terminology see supplementary Materials and Methods) We constructed a stochastic differential equation (SDE) model that retained the parameters of the ODE model but incorporated a description of intrinsic gene expression fluctuations, based on experimental measurements (supplementary Materials and Methods). In this model the amount of noise is defined by a value Ω, which we constrained based on experimental data. These constraints incorporated both the numbers of molecules in a cell and the amount of stochasticity suggested by the experimental measurements (supplementary Materials and Methods). Simulations with this model revealed that stochasticity in gene expression was sufficient to promote a switch from a pMN state to a p3 identity within the bistable region, and the probability of a noise-driven transition increased with higher levels of signal as the system approached the p3 monostable regime. Moreover, the hysteresis that is a consequence of the bistability (Panovska-Griffiths et al., 2013; Balaskas et al., 2012; Cohen et al., 2014) meant that transitions from pMN to p3 were more frequent than the reverse.

We used the SDE model to simulate a Pax6^−/−^ mutant. Compared with WT simulations, in the Pax6^−/−^ mutant, not only was the boundary of the Nkx2.2-expressing p3 domain displaced dorsally, but the boundary also showed markedly reduced precision (Fig. 1F-H). Thus, inclusion of intrinsic noise in the GRN dynamics was sufficient to accurately reproduce the alterations in the position and precision of gene expression boundaries.

### An Olig2 enhancer influences boundary precision

**Fig. 2.**
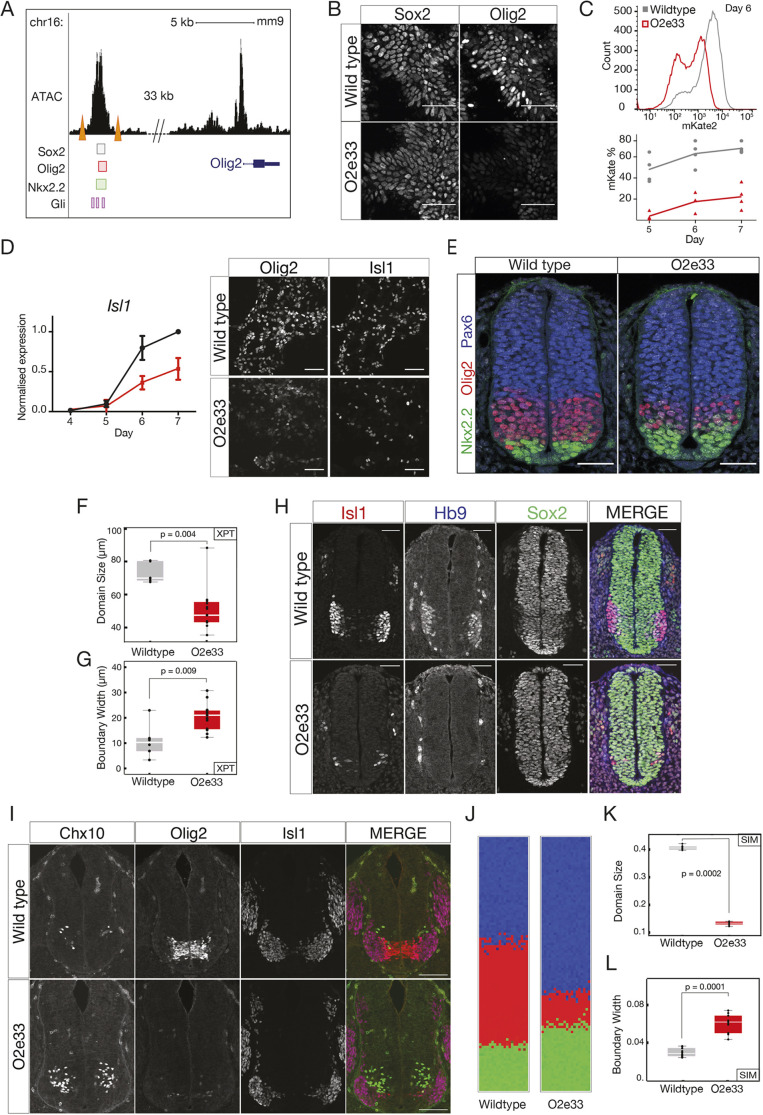
**An Olig2 enhancer affects precision of the pMN-p3 boundary.** (A) Chromatin accessibility (ATAC-seq) and predicted TF binding locations around Olig2. Orange triangles indicate the CRISPR target sites for deletion of the O2e33^−/−^ (Metzis et al., 2018; Kutejova et al., 2016; Peterson et al., 2012; Oosterveen et al., 2012). (B) Sox2 (expressed in all neural progenitors) and Olig2 at day 6 in neural progenitors differentiated from WT and O2e33^−/−^ ESCs exposed to 500 nM SAG. (C) Flow cytometry (top) for mKate2 fluorescence in Olig2-T2A-mKate2 ESC-derived neural progenitors exposed to 500 nM SAG. (D) RT-qPCR indicates that Isl1 is decreased in O2e33^−/−^ (red) cells compared with WT (black) cells differentiated under spinal cord conditions. Similarly, Olig2- and Isl1-expressing cells are reduced in mutant compared with WT. Data are mean±s.d. (E) Olig2, Pax6 and Nkx2.2 in transverse sections of E9.5 neural tube from WT and O2e33^−/−^ (red, Olig2; green, Nkx2.2). (F,G) Domain size and boundary width in WT (grey) and O2e33^−/−^ mutants (red). *n*=6 (WT), *n*=12 (O2e33), *P*=0.004. The p3-pMN boundary is wider in O2e33^−/−^ mutants compared with WT (*P*=0.009). (H) Isl1- and Hb9-expressing motor neurons are reduced in O2e33^−/−^ embryos compared with WT. (I) Chx10-expressing V2 neurons increase in the 02e33^−/−^ mutant. (J) Simulations of the O2e33^−/−^ model recapitulate *in vivo* observations of a narrower pMN domain and decreased precision of the p3-pMN boundary. (K,L) Boundary width (I) and position (H) from simulations. *n*=10 (WT), *n*=10 (O2e33), *P*=0.0001. Box plots show upper and lower quartile and mean. Statistical significance was determined using a Mann–Whitney test. Scale bars: 50 μm (B,D,E,H); 100 μm (I).

To test the hypothesis that the regulatory dynamics of the GRN affect the precision of patterning, we sought to alter the strength of interactions within the network. We turned our attention to the cis-regulatory elements controlling the TFs in the GRN, as regulatory elements have been shown to affect the reliability of patterning in other systems (Perry et al., 2011; El-Sherif and Levine, 2016). Several predicted regulatory regions are located in the vicinity of Olig2; these include a prominent candidate region 33 kb upstream of the Olig2 gene (Oosterveen et al., 2012; Peterson et al. 2012), which we termed O2e33. This region binds (1) the repressor Nkx2.2; (2) Sox2, which activates Olig2; and (3) the Gli proteins, the transcriptional effectors of the Shh pathway (Fig. 2A) (Oosterveen et al., 2012; Peterson et al., 2012; Nishi et al., 2015; Kutejova et al., 2016), and becomes accessible in neural progenitors (Metzis et al., 2018). To test the role of O2e33 in the network, we first analysed its function *in vitro* in neural progenitors differentiated from mouse embryonic stem cells (ESCs) Gouti et al. (2014). Unlike WT cells, which express high levels of Olig2 at Day 5 of differentiation [Gouti et al. (2014); Sagner et al. (2018)], cells in which the O2e33 enhancer had been deleted (O2e33^−/−^) had a marked reduction in levels of Olig2. By Day 6, Olig2 expression had increased in O2e33^−/−^ cells, but the percentage of cells and the level of expression never reached that of WT (Fig. 2B,C). Consistent with the role of Olig2 in the generation of motor neurons (MNs), the production of these neurons was substantially decreased in O2e33^−/−^ cells (Fig. 2D).

We used the experimentally observed delay in Olig2 induction to identify changes in model parameters that mimic the effect of deleting the O2e33 enhancer. This resulted in a reduction, but not the elimination, of the parameters that described the regulation of Olig2 by TFs that have been shown experimentally to bind O2e33 (supplementary Materials and Methods). Of the parameter sets that delayed Olig2 induction *in silico*, most predicted the generation of a smaller pMN domain, resulting from a ventral shift in the dorsal boundary. Strikingly, many of the parameter sets also predicted a loss of boundary sharpness of the p3-pMN boundary. To test these predictions, we generated mutant mice lacking the O2e33 enhancer (Materials and Methods). Assaying the neural tube of embryos from these mice revealed lower Olig2 expression levels in pMN cells and a delay in the induction of Olig2 in O2e33^−/−^ embryos compared with WT, in agreement with *in vitro* results (Figs S2, S3). As predicted by the *in silico* analysis, the pMN domain was decreased in size in O2e33^−/−^ embryos, with its dorsal limit of expression noticeably more ventrally positioned (Fig. 2E). Moreover, the boundary between the pMN and p3 domain was less precise than in WT (Fig. 2E-G). Consistent with the reduced domain size, there was a significant reduction in the generation of MNs in O2e33^−/−^ embryos and a concomitant increase in V2 neuron production (Fig. 2H,I). The decrease in the precision of the boundary, despite continued expression of Olig2 and Pax6 in pMN cells, suggests that secondary correction mechanisms, such as cell-cell adhesion or cell sorting, do not suffice to determine the precision of the boundary between these two domains.

Using the *in vivo* observations, we further constrained the parameter space of the model by restricting our analysis to parameter sets that generated an imprecise p3-pMN boundary and altered the position of the pMN-p2 boundary (supplementary Materials and Methods). This produced simulations in which the loss of boundary precision in the O2e33^−/−^ embryos is not as severe as the Pax6^−/−^ phenotype, in line with the experimental data (Fig. 2J), and the width of the boundary and the size of the pMN domain were consistent with *in vivo* analysis (Fig. 2K,L). Taken together, the data suggest that in the absence of Pax6 function or the activity of the O2e33 enhancer, stochasticity of gene expression decreases the precision of the p3-pMN boundary.

### Rate of stochastic switching is controlled by GRN dynamics

**Fig. 3.**
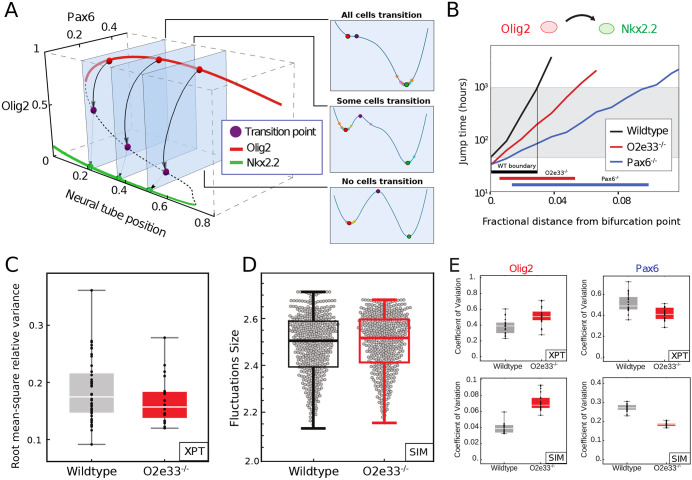
**The rate of transition between progenitor states is determined by the GRN structure.** (A) A three-dimensional bifurcation diagram illustrates bistability for pMN (red; expressing Olig2 and Pax6) and p3 (green; expressing Nkx2.2) with a transition point (unstable fixed point of dynamics, purple). Noise-driven transition pathway (formally, MAP) from pMN to p3 is indicated by black arrows. Right panels: Conceptual representations of the transitions as one-dimensional Waddington landscape sketches. (B) Fate jump times calculated from simulations: pMN to p3 in WT (black), Pax6^−/−^ (blue) and O2e33^−/−^ mutants (red). Fractional distance refers to distance from the bifurcation point. Grey shading indicates where transitions can occur on developmental timescales. (C) Total variance in gene expression per embryo (Olig2 and Pax6) within the pMN domain for WT (grey) and O2e33^−/−^ embryos (red). Relative root-mean-square variance of WT and O2e33^−/−^ embryos captures the total noise of the system. There was no significant change in the noise levels between genotypes (*P*>0.05). (D) Measurements of noise *in silico* in the pMN domain in WT and O2e33^−/−^. Each grey point represents an individual configuration (supplementary Materials and Methods, *P*>0.05). (E) CV for Olig2 (left) and Pax6 (right) in WT (grey) and O2e33^−/−^ (red) from experimental data (top) and *in silico* simulations (bottom). Box plots show upper and lower quartile and mean. Statistical significance was determined using a Mann–Whitney test.

To understand the mechanism by which Pax6 and O2e33 contribute to boundary precision, we explored the dynamical properties of the SDE model. The model did not predict a difference in the magnitude of the fluctuations in the expression of individual genes between the WT and the Pax6 mutant (supplementary Materials and Methods). Consistent with this, experimental measurements of the coefficient of variation (CV) of Olig2 from WT and Pax6^−/−^ embryos did not reveal significant differences (Fig. 1I). This raised the possibility that, rather than the size of fluctuations in individual genes, the change in precision was a consequence of the regulatory interactions of the network. The model of the GRN predicts a bistable regime between the two steady states of Nkx2.2 (p3) and Olig2/Pax6 (pMN) (Fig. 3A) (Balaskas et al., 2012; Cohen et al., 2014). In the absence of noise, the transition between the two steady states is determined solely by the level of Shh signalling. However, in the presence of intrinsic noise, fluctuations in gene expression can result in spontaneous transitions between pMN and p3 identity within the bistable region (Perez-Carrasco et al., 2016). Below a threshold of Shh signalling, the rate of transitions is very low and cells remain in the pMN state. Conversely, above a certain level of Shh signalling, transitions from the pMN to the p3 steady state take place so rapidly that almost all cells undergo this transition. In between these two regimes, a region of heterogeneity is observed in which there is an intermediate probability for each cell to transition spontaneously (≤50 h, see Fig. 3A,B). We calculated the characteristic time for transitions between the pMN and p3 states at different dorsal-ventral positions of the neural tube. We termed this ‘fate jump time’. For WT, fate jump time changes rapidly in response to Shh signalling, implying that there is only a limited region in which the effective probability of transitions is not 0 or 1 (Fig. 3B, black line). By contrast, the larger region of heterogeneity observed in the Pax6^−/−^ mutant is due to the weaker dependence of fate jump time on levels of Shh signalling (Fig. 3B; blue line). There is a larger range of Shh levels for which noise driven transitions are possible and, therefore, a larger boundary region in which cells in both p3 and pMN states exist.

Fate jump times changed more slowly for O2e33^−/−^ than for WT (Fig. 3B), but more rapidly than for the Pax6^−/−^ system. This is in line with the boundary precision of O2e33^−/−^ embryos falling between that of WT and Pax6^−/−^. Analysis *in vivo* of the magnitude of the combined fluctuations in Pax6 and Olig2 indicated that it was similar in WT and O2e33^−/−^ (Fig. 3C; Materials and Methods). Consistent with this, the combined magnitude of fluctuations of Pax6 and Olig2 in simulations were similar in WT and O2e33^−/−^ mutants. This suggested that, similar to Pax6^−/−^ embryos, the decreased precision was not the result of an increase in the overall magnitude of fluctuations (Fig. 3D; Fig. S9). In addition, simulations of the O2e33^−/−^ mutant predicted that variability in Olig2 should increase, whereas the variability of Pax6 should decrease. In agreement with this, the CV of Olig2 and Pax6 between WT and O2e33^−/−^ *in vivo* were increased and decreased, respectively (Fig. 3E).

### An effective one-dimensional dynamical landscape determines boundary precision

To investigate the reasons for the change in fate jump time in O2e33^−/−^ and Pax6^−/−^ mutants, we explored the effect of these perturbations on the dynamics of the system (see supplementary Materials and Methods). We stress that the dynamics in our computational model are not formally derived from the gradient of an energy landscape; it is only in one dimension that such a representation can always be constructed. Nonetheless, the landscape picture can provide a useful metaphor for thinking about transitions between fixed points. More importantly, we show below that it can be made precise as an effective one-dimensional landscape along the typical trajectories that gene expression levels follow in such transitions.

**Fig. 4.**
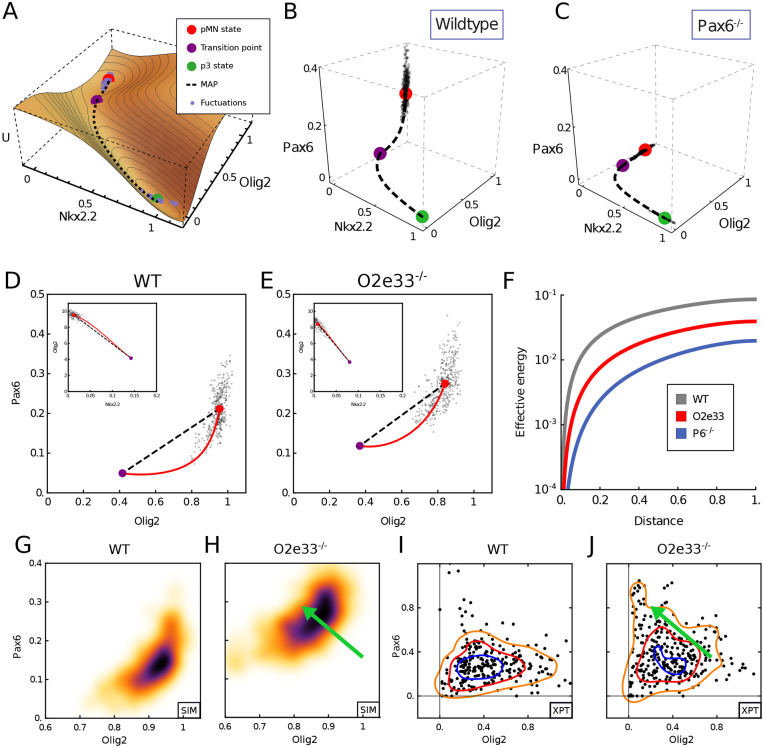
**Mutant phenotypes affect the configuration of gene expression fluctuations.** (A) A quasi-potential (U) representation of the neural tube dynamical system in the region in which noise-driven transitions result in heterogeneity between pMN and p3 states. The landscape is a sketch to aid visualisation of the MAP, which is directly calculated from the system. (B,C) Gene expression space view of the transition path from pMN (red point) to p3 (green point) steady states via the transition point (purple point). Simulated trajectory (dots) shows stochastic fluctuations from the pMN steady state. Axes show relative expression levels. WT (left) and Pax6^−/−^ (right) for neural tube position at fraction 0.1 of total neural tube length dorsal to the bifurcation point. (D,E) Projection into Olig2-Pax6 gene expression space of the MAP (red) predicted from the model and simulated trajectory (dots) in WT (I) and O2e33^−/−^ (J) at the same position as G and H. Insets show projection onto Nkx2.2-Olig2 axes. (F) Effective energy barrier (cumulative action) for noise-induced transitions, plotted along the transition path (normalised to unit length) at the same neural tube positions as G and J. WT (grey) has a higher barrier than O2e33^−/−^ (red), leading to longer jump times; O2e33^−/−^ in turn has a higher barrier than Pax6^−/−^ (blue). (G,H) Simulated Pax6 and Olig2 expression levels (black dots) for WT and O2e33^−/−^ in regions proximal to the p3-pMN boundary. A shift to lower Olig2 and higher Pax6 for O2e33^–/–^ can be observed (green arrow). (I,J) A shift to higher levels of Pax6 and reduced levels of Olig2 is observed in cells from O2e33^−/−^ mutants *in vivo* compared with controls. Axes show fluorescence intensity (arbitary units). Contour lines correspond to densities of the distribution of points, 0.6 (orange), 1.6 (red) and 2.6 (blue).

Transitions between p3 and pMN states involve the system passing through, or very close to, a point in gene expression space that is characterised by specific levels of the TFs; we refer to this as the ‘transition point’ (Fig. 4A-C; purple point, also known as an unstable fixed point). In the dynamical systems literature this is denoted a saddle point (Fig. 4A). Simulations of the SDE model indicated that intrinsic fluctuations around the pMN state are initially directed away from the transition point in WT. By contrast, in the Pax6 mutant fluctuations are oriented directly towards the transition point. As a consequence, fluctuations of the same magnitude would be more likely to reach the transition point in Pax6^−/−^ than WT cells. To characterise this rigorously, we calculated the most likely gene expression trajectory that a stochastic transition caused by fluctuations in gene expression will take between the pMN and p3 steady states. This path is obtained as the minimum of an ‘action functional’ – the minimum action path (MAP, see supplementary Materials and Methods). Intuitively, this is the path of least resistance, which noise-driven transitions are therefore most likely to follow in gene expression space. Motion along this path provides a one-dimensional portrait of the dynamics in a noise-induced transition, and is an analytical representation of the behaviour that can be observed in simulations. We can define an effective energy along the MAP, and the maximum of this energy at the transition point is the effective energy barrier that governs the jump time of the system (Fig. 4A; supplementary Materials and Methods) (Perez-Carrasco et al., 2016; Kleinert, 2009; Bunin et al., 2012). Consistent with the SDE simulations, in WT, the MAP from the pMN to p3 steady state does not follow the shortest route leading to the transition point. Instead, the levels of Pax6 drop rapidly and pitch away from the transition point, resulting in the most likely gene expression path between steady states to be curved (Fig. 4B). By contrast, in the absence of Pax6, the MAP is directly oriented towards the transition point (Fig. 4C). Taken together, the analysis suggests that the GRN affects the precision of a domain boundary by determining the dynamics of transitions between stable fixed points and the corresponding effective energy landscape, without changing the level of noise in overall gene expression. For the O2e33^−/−^ mutants the MAP from pMN to p3 curved away from the shortest path to a lesser extent than for the WT; stochastic simulations further confirm this behaviour (Fig. 4D,E). Thus, in the absence of the O2e33 enhancer, stochastic fluctuations around the pMN steady state tended to take the system closer to the transition point than similar magnitude fluctuations in WT, making a noise-driven switch in fate more likely in the mutant. Nevertheless, the curvature in the path in the O2e33^−/−^ system was greater than in the Pax6^−/−^ system, providing an explanation for the greater imprecision in Pax6^−/−^ embryos compared with the O2e33^−/−^ mutant (Fig. 4B-E).

To explore this phenomenon further, we calculated the action along the path for each genotype (de la Cruz et al., 2018) (Fig. 4F; Materials and Methods). This represents the effective energy required to reach a point along the transition path and is a measure of the extent of the barrier that has to be overcome for a fate transition. Consistent with the results of the simulations, the effective energy necessary for a noise-induced transition was greatest for WT, less for O2e33^−/−^, and lowest for the Pax6^−/−^ mutant. Moreover, the analysis indicated that the initial part of the trajectory presented a more significant barrier to noise-induced transitions in the WT than O2e33^−/−^ and Pax6^−/−^ mutants (Fig. S6A), corresponding to the relative divergences of their transition trajectories from the shortest route to the transition point. This also highlights further that the loss of boundary precision is not a simple result of reduced dimensionality of the system, as the O2e33^−/−^ mutant retains expression of all TFs.

An experimentally testable signature of the alteration in the dynamical landscape in O2e33^−/−^ mutants would be changes in the relative expression levels of Olig2 and Pax6 in individual cells. In cells close to the pMN-p3 boundary, O2e33^−/−^ mutants are predicted to have higher levels of Pax6 and lower levels of Olig2 than WT (Fig. 4G,H). We therefore compared single cell immunofluorescence in the boundary region of WT and O2e33^−/−^ embryos (Fig. 4I,J; Materials and Methods). Consistent with the predictions, O2e33^−/−^ mutants had higher levels of Pax6 and lower levels of Olig2 than WT. Thus, the experimental evidence supports the idea that the strength of regulatory interactions encoded in the GRN contributes to the precision of domain boundaries by configuring the effective dynamical landscape of the system to reduce the likelihood of a stochastic fluctuation resulting in a noise-driven change in cell identity.

### A computational screen identifies mechanisms for precise boundaries

**Fig. 5.**
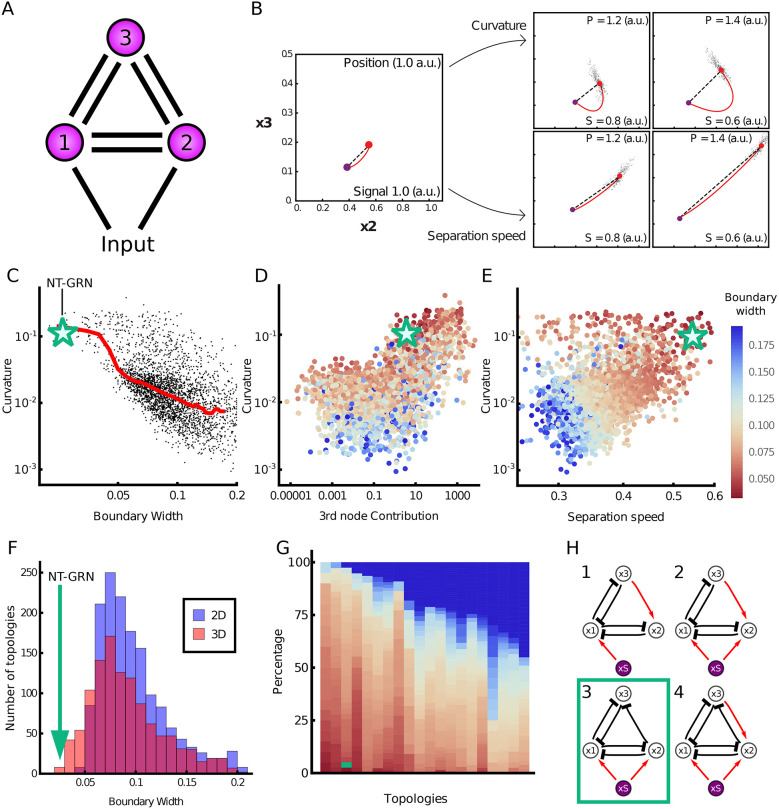
**Computational screen reveals the design principles of precision.** (A) Three-node networks, comprising all possible interactions and a morphogen input into two nodes. (B) Two mechanisms for producing a precise boundary. Close to the boundary (position 1.0 a.u.; signal 1.0 a.u.) the steady state (red point) is near the transition point (purple point) in gene expression space. Further away (increasing position; decreasing signal), curvature of the MAP (red line) with respect to the shortest pathway (top row), or the rate at which the steady state separates from the transition point (bottom row), can contribute to increasing boundary precision. (C) For each network recovered from the screen (points), the boundary width was compared with the deviation of the MAP from the shortest path to the transition (curvature). Median value (red line) illustrates that sharper boundaries (smaller width) tend to have higher MAP curvature. The green star represents the WT neural tube network. (D) Curvature compared with the effective contribution of the third node in the network (boundary width indicated by the colour of the point). (E) Curvature compared with signal sensitivity. The colour of the points by boundary width indicates that both high curvature and high signal sensitivity contribute to the sharpest boundaries. (F) Histogram of boundary width in three-dimensional (red) and two-dimensional (blue) networks. The green arrow represents the WT network. (G) The most common topologies, arranged in order of the fraction of networks with precise boundaries; each column represents an individual topology. Dark blue indicates networks with a wider boundary. Topologies are shown in Fig. S16. (H) Four topologies that favour the sharpest boundaries. These networks comprise inhibition from node 2 to node 3, and lack repression from node 3 to node 2. The WT neural tube network has topology 3 (green box). a.u., arbitrary units.

To investigate whether other mechanisms could affect boundary precision, we performed a computational screen to identify three-node networks capable of generating a sharp boundary in response to a graded input (Fig. 5A; supplementary Materials and Methods). For the networks recovered from the screen, we compared the boundary precision with the extent the MAP deviates from the shortest path to the transition, a quantity that we refer to as ‘curvature’ (supplementary Materials and Methods). This showed a positive correlation, consistent with our observations in the WT network, of high curvature and low boundary width. Although there is no general principle that forces transition trajectories to follow the shortest path, we found that networks with trajectories that align more closely to the shortest path tend to produce less precise boundaries. This correlation supports the idea that the shape of the transition pathway contributes to boundary precision, and precision is not simply a result of changes in dimensionality (Fig. 5C). Nevertheless, for any given level of boundary sharpness, there were a range of MAP curvature values. We therefore investigated additional features that might affect boundary precision. We found a subset of the networks do not rely on path curvature to achieve precision and instead functioned effectively as two-node networks (Fig. 5D). For these networks, the major contributor to boundary precision was the rate at which the steady state and transition point separated in response to changes in the level of the input signal: the higher the rate of separation, the sharper the boundary (Fig. 5B). We termed this ‘signal sensitivity’. The most precise boundaries were generated by networks that exploited both signal sensitivity and curvature through the use of all three nodes, which includes the Pax6-Olig2-Nkx2.2 network (Fig. 5E,F).

Finally, we assessed whether particular network topologies favoured boundary sharpness. Many topologies were able to generate sharp boundaries (Fig. 5G,H; supplementary Materials and Methods), but four topologies appeared to be most effective (Fig. 5H). These tended to have similar signal sensitivities but much higher curvature than the networks with other topologies (Fig. S17). Crucial for this behaviour was the inhibition of *x*_3_ by *x*_2_ and the absence of repression of *x*_2_ by *x*_3_ (Fig. 5G; Fig. S16). This regulatory configuration generates curvature by allowing a steep decrease in *x*_3_, while sustaining high levels of *x*_2_ before the transition. Hence, an understanding of the dynamical properties of the GRN offers an explanation for its structure and the resulting gene expression behaviour that determines tissue patterning.

## DISCUSSION

In this study, we provide evidence that the spatial heterogeneity that results from the stochasticity of gene expression can be attenuated by the dynamics of the GRN to enhance the precision of gene expression in developing tissues. This mechanism does not rely on suppressing stochastic fluctuations in individual genes, or on cell-to-cell communication, but instead configures the dynamical landscape of the regulatory network to increase the fidelity of decision making. This strategy – ‘precision by design’ – highlights the capacity of gene regulatory circuits to contribute to robust tissue patterning, and identifies a mechanism that might be exploited in other biological settings requiring precise responses from groups of cells.

### GRN dynamics contribute to precise boundaries without attenuating gene expression noise

Molecular noise is a universal feature of gene expression (Raj and van Oudenaarden, 2008; Raser and O'Shea, 2005; Chalancon et al., 2012). Despite this, patterns of gene expression in developing tissues are remarkably reproducible and precise, as exemplified by the sharp boundaries of gene expression that delimit distinct domains of cells in many tissues. This spatial precision is crucial for the accurate assembly of tissues. For example, along the anterior-posterior axis of the *Drosophila* embryo, the expression of genes that partition the blastoderm into the major elements of the body axis are positioned with an accuracy of 1% or better (Dubuis et al., 2013; Petkova et al., 2019). Similarly, in the central nervous system the correct positioning of different neuronal subtypes is a major determinant of their subsequent patterns of connectivity and underpins the formation of functional neural circuits (Jessell et al., 2011; Balaskas et al., 2019).

Mechanisms involving cell-cell interactions to correct initial imprecisions in the spatial organisation of tissues have received considerable attention (Xu et al., 1999; Standley et al., 2001; Rudolf et al., 2015; Dahmann et al., 2011; Addison et al., 2018). Differential cell adhesion between neural progenitors with different cellular identities has been proposed to refine initially disordered patterns (Lei et al., 2004; Xiong et al., 2013; Tsai et al., 2020). However, neither differential adhesion nor cell sorting appear to be the sole explanation for the precision of patterning in the neural tube. Lineage tracing in the mouse and chick neural tube (Kicheva et al., 2014; Leber and Sanes, 1995) indicates that sister cells form contiguous clones and there is no evidence that clones at a domain boundary behave in a way compatible with differential interactions across a boundary. Moreover, in both Pax6^−/−^ and O2e33^−/−^ mutants, neural progenitors with distinct identities, pMN and p3, producing MNs and V3 neurons, respectively, continue to be generated, but these different progenitor types intermix to a greater extent than normal. If the differential expression of cell adhesion molecules or different mechanical properties explained the sharpness of the boundary between pMN and p3 cells, this would lead to the sorting of pMN and p3 cells in the mutant embryos. Nevertheless, cell adhesion might play a role in the neural tube of teleosts (Xiong et al., 2013; Tsai et al., 2020). Unlike the epithelial neural tube of amniotes, the zebrafish neural tube initially comprises unpolarised non-epithelial cells and sister cells disperse widely, including contralaterally, in the neural tube. This raises the possibility that differential cell adhesion plays a more important role in the anamniote neural tube.

Similar to many developing tissues, the neural tube is patterned by graded signals that are transformed into discrete cell identities by the downstream GRN acting as a series of toggle switches to produce discontinuous changes in cell identity across the tissue (Sagner and Briscoe, 2019). Previous studies have explored how properties of extracellular patterning signals (Bollenbach et al., 2008; Tkačik et al., 2015; Lucas et al., 2018; Sokolowski et al., 2012; Zagorski et al., 2017) and features of the regulation of individual genes (Perry et al., 2010; Frankel et al., 2010; Lagha et al., 2012; Little et al., 2013; Battich et al., 2015; Dickel et al., 2018; Osterwalder et al., 2018; Paliou et al., 2019) can contribute to the fidelity of gene expression. Some of these mechanisms may play a part in the precision of neural tube gene expression. For example, paralogs of several of the key TFs are co-expressed in the neural tube and appear to function, at least partially, redundantly (Vallstedt et al., 2001; Holz et al., 2010). In addition, the provision of antiparallel signalling gradients emanating from the opposing dorsal and ventral poles of the neural tube have been implicated in increasing the precision of gene expression in central regions of the spinal cord (Zagorski et al., 2017). However, the ventral regions of the neural tube, in which the p3-pMN boundary is positioned, are out of range of the dorsal signal. The changes in boundary precision in the neural tube of the Pax6^−/−^ and O2e33^−/−^ mutants are not explained by changes in noise amplitude in individual genes or global changes in the magnitude of the noise. Instead, the genetic perturbations we analysed alter the dynamics of the GRN, and these change the configuration of gene expression fluctuations and make noise-driven transitions between cell states more likely. Thus, the dynamics of the GRN affect patterning precision, without altering the stochasticity of individual components of the system, indicating that the configuration of gene expression noise, not simply the magnitude, affects development precision.

Stochastic fluctuations in gene expression are expected to result in variations in the position at which cells switch identity and produce indistinct boundaries. There is a trade-off between the steepness, precision and speed of boundary formation (Chalancon et al., 2012; Lv et al., 2014; Perez-Carrasco et al., 2016; Tran et al., 2018). If gene expression was deterministic, a graded signal controlling such a switch would generate a sharp precisely positioned gene expression boundary in the tissue. However, the effect of stochastic fluctuations is that an increase in non-linearity and switch-like behaviour decreases boundary precision: stochastic fluctuations generate a change in gene expression that is independent of changes in signal input. Our analysis of the Pax6-Olig2-Nkx2.2 network revealed that the GRN is configured to decrease the probability of such spontaneous noise-driven transitions while retaining the ability to produce discontinuous switch-like changes in gene expression, thereby generating a sharp precise boundary in the tissue. This mechanism enhances boundary precision even in the presence of noise in the signalling gradient (supplementary Materials and Methods; Fig. S18). Moreover, the same regulatory mechanism that decreases the probability of a noise-driven transition from pMN to p3 also produces hysteresis. This ratchet-like effect means that once a cell has adopted a p3 identity it is unlikely to transition back (Balaskas et al., 2012). Thus, the dynamics of this GRN increase the precision of the pMN-p3 boundary by decreasing the probability of transitions between pMN and p3 in either direction. As a consequence of the dynamics of the system, the characteristic wave of gene expression associated with neural tube patterning can be halted by cutting short exposure to Shh. This means that to establish the desired pattern, Shh input into the GRN must be sustained over time, resulting in cells being dependent on both Shh levels and duration of exposure (Balaskas et al., 2012).

### Configuring the dynamical landscape to maximise precision

Viewed from the perspective of the Waddington landscape analogy (Waddington, 1957), spontaneous changes in cell state resulting from gene expression fluctuations would be represented as a cell being displaced from one valley to another by traversing the intervening ridge (supplementary Materials and Methods). The dynamical landscape produced by the Pax6-Olig2-Nkx2.2 network is configured so that the height of the ridge between the two valleys changes rapidly as the level of morphogen signalling changes. This is evident from analysis of the MAP, which reveals that transition trajectories between cell states diverge substantially from the shortest route to the transition point (Fig. 4B-E). The consequence of this is that the effective energy necessary for a noise-induced fate transition was higher for WT than either of the mutants with a perturbed GRN (Fig. 4F; supplementary Materials and Methods). Thus, the GRN minimizes the range of signalling in which noise-induced transitions are likely to occur, without altering the stochasticity of individual genes, hence increasing boundary sharpness.

This mechanism, which we termed ‘curvature’, was identified in an unbiased computational screen of three-node networks responding to a graded input signal (Fig. 5). In addition, the screen recovered a second mechanism – ‘signal sensitivity’ – that relied on the rate at which the two cell states separated in response to changes in the level of input signal (Fig. 5B). In the context of the Waddington landscape, signal sensitivity can be viewed as small changes in signal levels producing large changes in the distance between the two valleys. A feature of this second mechanism is that it can be implemented with only two genes. However, instead of producing two cell states both with uniform levels of gene expression, one of the resulting cell states is characterised by a gradient of gene expression (Fig. S15). This might limit its utility in some tissue patterning roles. By contrast, the curvature mechanism requires a minimum of three nodes to implement, but it is able to produce two cell states with almost constant levels of gene expression. Nevertheless, the two mechanisms of speed and curvature are not mutually exclusive, and the networks recovered by the screen that generated the most precise boundaries combined both mechanisms. Key to identifying these properties was performing a screen with three, and not two, species, as the extra dimension allows for curvature to arise. These properties would also be relevant with networks containing more than three species, and it will be interesting to investigate whether there are additional more complex behaviours in higher dimensions that can contribute to the precision of gene expression patterns.

### Regulatory principles of patterning precision

Similar to other recent studies (Cotterell and Sharpe, 2010; Schaerli et al., 2014; Verd et al., 2019), the screen indicated that the dynamics of the networks, not simply the network topology, were key to determining the resulting precision. A feature shared by many of the networks with the sharpest boundaries, including the neural tube network, was an asymmetry in inhibition between two of the genes (Fig. 5H). Specifically, *x*_2_ (Olig2) repressed *x*_2_ (Pax6), but not vice versa. Moreover, the graded expression of Pax6 (*x*_3_) within the domain is indicative of the signal sensitivity mechanism, providing evidence that this too contributes to boundary precision while allowing uniform levels of Olig2 expression (*x*_2_; the gene necessary for defining the identity of this domain). This analysis therefore raises the possibility that the dynamics of the Pax6-Olig2-Nkx2.2 network were adopted in the developing vertebrate neural tube for its capacity to generate distinct cell-type identities with precise boundaries. In this context, it is striking that gene circuits with similar structure and dynamics have been implicated in the patterning of the anterior-posterior axis of the *Drosophila* embryo (Akam, 1987; Ingham, 1988; Sánchez and Thieffry, 2001; Manu et al., 2009; Verd et al., 2017) and the *Drosophila* eye imaginal discs (O'Neill et al., 1994; Rebay and Rubin, 1995; Graham et al., 2010) (supplementary Materials and Methods, Figs S19, S20). Taken together therefore, the computational screen defines design features of multistable gene circuits that are suited to the generation of sharp boundaries in response to graded inputs.

The dynamics of a GRN are governed by the strength of regulatory interactions between the components of the network, which in turn are determined by cis regulatory elements and their binding to TFs (Davidson, 2010). Many genes involved in development are associated with two or more cis regulatory elements that seem to function in a partially redundant manner (Perry et al., 2011; El-Sherif and Levine, 2016; Cannavò et al., 2016; Dunipace et al., 2019). This appears to be the case for Olig2, in which removal of the O2e33 element perturbs, but does not completely abrogate, Olig2 expression (Fig. 2). This supports the idea that one function of multiple cis regulatory elements is to provide robustness and precision to gene expression (Perry et al., 2010; Frankel et al., 2010; Lagha et al., 2012; Battich et al., 2015; Dickel et al., 2018; Osterwalder et al., 2018; Paliou et al., 2019; Tsai et al., 2019). Our analysis indicates that these functions are not simply a consequence of multiple elements supplying duplicate activities. Instead, individual cis regulatory elements provide specific dynamical properties to gene regulation that sculpt the gene expression landscape. Thus, distinct cis regulatory elements of a target gene serve specific dynamical functions within a GRN.

Taken together, our analysis illustrates how a tissue level feature, the spatial precision of gene expression patterns, is influenced by cell-autonomous mechanisms, implemented by cis regulatory elements that influence the activity of a network of interacting TFs. The data reveal that the potential detrimental effects of stochastic fluctuations in gene expression that would lead to spatial heterogeneity can be attenuated by the dynamics of the GRN. We term the strategy ‘precision by design’ as it arises from the integrated function of the gene circuit and is not intrinsic to any individual network component. This provides insight into decision making in multicellular systems and highlights how an understanding of the dynamics of GRNs can explain its structure and function. More generally, identifying the principles that produce robust and precise outputs despite the inherent stochasticity of gene expression should assist in the future design, modification and engineering of gene circuits.

### MATERIALS AND METHODS

#### Mouse strains

Mouse strains containing the following alleles were used: Pax6^Sey^ (Ericson et al., 1997) and O2e33^−/−^ in strain backgrounds C57BL/6Jax and F1(B6xCBA), respectively. The O2e33^−/−^ allele was derived using zygote injection of CRISPR gRNA and Cas9 plasmids (see below). Embryos were transferred to pseudopregnant females and subsequent pups were genotyped. O2e33^−/−^ mice were maintained as a heterozygous population; the line was sub-viable with less than 2/40 homozygous offspring surviving. Embryos for analyses were collected at the indicated time points following a mating, with the day of plug detection designated E0.5. All animal procedures were carried out in accordance with the Animal (Scientific Procedures) Act 1986 under the Home Office project licence PPL80/2528 and PD415DD17.

#### Embryonic stem cell culture

For the enhancer deletion *in vitro*, mouse ESCs containing a fluorescent reporter co-translated with Olig2 (Olig2::T2A-mKate2) (Sagner et al., 2018) were used. Mouse ESCs were maintained on mitotically inactivated fibroblasts (feeder cells) in ES medium with 1,000 U/ml leukaemia inhibitory factor. Cells were differentiated to spinal cord neural progenitors as described previously (Gouti et al., 2014). To initiate differentiation, ESCs were dissociated using 0.05% Trypsin (Gibco) and panned in ES medium on culture plates for 2×15 min to remove feeder cells. ESCs were collected, spun down and resuspended in N2B27 medium. A total of 50,000 cells were plated on 35mm CellBIND dishes (Corning). Dishes had been coated with 0.1% gelatine in PBS before addition of 1.5ml of N2B27 with 10 ng/ml bFGF. After 48 h, medium was replaced with N2B27 plus 10ng/ml bFGF plus 5uM CHIR99021 (Axon). After 24 h, at D3, medium was replaced with N2B27 plus 100nm RA (Sigma-Aldrich) and 500nm Smoothened agonist (SAG, Calbiochem). This was repeated every 24 h.

#### CRISPR/Cas9 targeting

For CRISPR/Cas9-mediated excision of the 33 kb enhancer, two pairs of short guide RNA (sgRNA) sequences were designed to target either side of the enhancer region. The ZiFit online tool (http://zifit.partners.org/) was used to select guides that had the lowest number of potential off target sites. sgRNA sequences (ACTTTGTAAGCCGAGCC) and (GATAATCGCCTCCCTCC) were cloned into pX459 v2.0 (Addgene, Ran et al., 2013) and transfected into ESCs via nucleofection. This generated a cell line with a 995 bp deletion (chr16: 91192464-91193458). Two separate clones were analysed to determine whether there was substantial clonal variation. A second line was generated with a larger deletion of approximately 3.3kb using sgRNA sequences (GTTTATGGCTCATCCCC and TCCAGGCTCCCATATCC). Cell lines with this larger deletion yielded the same results as the smaller deletion (Exelby, 2020). To generate the mouse line, plasmids encoding the sgRNAs for the 3.3kb deletion were injected into zygotes before being transferred to pseudopregnant females. The mouse line generated had a 3259 bp deletion (chr16: 91191295-91194570).

To assess Olig2 protein copy number, a transgenic cell line was constructed, Olig2-HASnapTag. Sequence encoding an HA-tagged SnapTag was placed at the C-terminus of the endogenous coding sequence for Olig2 via homologous recombination using CRISPR. The SnapTag sequence was extracted from the pSNAPf vector (New England Biolabs, N9183S) and inserted into a plasmid containing Olig2 and targeted as described previously (Sagner et al., 2018).

#### Protein copy number quantification

The concentration of recombinant proteins (used as standards) was calculated from Coomassie staining (GelCode Blue Stain Reagent, Thermo Fisher Scientific). Recombinant proteins used were Pax6 (Bioclone, PI-0099), Nkx2.2 (MyBioSource, MBS717917) and SnapTag (New England Biolabs, P9312S). A solution of SNAP-tag (5 μm) was labelled with Janelia Fluor JF549 (TOCRIS, 6147) SnapTag Ligand at 10 μm (assembled in house) for 30 mins at 37°C.

To determine Pax6 and Nkx2.2 average molecule number per cell, a WT HM1 mouse ESC line was used (Doetschman et al., 1987). Cells were lysed in RIPA buffer supplemented with protease inhibitors. The cell lysates were analysed by western blot, with lysate from a known number of cells loaded per lane. The following antibodies were used: rabbit anti-Pax6 (Millipore, AB2237, 1:2000), mouse anti-Nkx2.2 [Developmental Studies Hybridoma Bank (DHSB), 745A5, 1:50], donkey anti-mouse IRDye 800CW (Licor) and donkey anti-rabbit IRDye 680RD (Licor). Blots were scanned using an Odyssey Scanner (Licor). We used the cell line Olig2-HA-SnapTag to determine protein copy number for Olig2. Cells for Olig2 and Nkx2.2 copy numbers were differentiated as described previously. For Pax6, cells were exposed to 100 nm retinoic acid only from day 4 to induce a more dorsal spinal cord cell fate. One day before sample collection, the cells were incubated with Janelia Fluor JF549 SnapTag Ligand (assembled in house) directly in the medium at 1 μM overnight. Cells were then lysed in RIPA buffer supplemented with protease inhibitors. A known number of cells were loaded per lane. Gels were scanned using Typhoon FLA 9500. To determine the percentage of expressing cells, flow cytometry was carried out as described in the flow cytometry section.

#### Flow cytometry analysis

Cells were dissociated using 0.05% trypsin and collected in ES medium. Cells were then washed in PBS and resuspensed in PBS containing live-cell Calcein Violet dye (Life Technologies). Control and O2e33^−/−^ cells were differentiated in parallel and analysed together. Control cells differentiated without SAG from day 4 were used to set population gates for mKate^+^ cells.

For protein quantifications, flow cytometry was used to determine the percentage of cells expressing Olig2, Pax6 and Nkx2.2. Cells were labelled with either PE Mouse anti-Nkx2.2 (BD Pharmingen, 564730, 1:20), Alexa Fluor 647 mouse anti-Human Pax6 (BD Pharmingen, 562249, 1:50), goat anti-Olig2 (R&D Systems, AF2418, 1:800) or donkey anti-goat 405 (Biotium, 20398, 1:500). Flow analysis was performed using a Becton Dickinson LSRII flow cytometer.

#### qPCR assays

The mRNA was extracted using an RNeasy Mini Kit (Qiagen) according to the manufacturer's instructions. A volume of 1 μg RNA was used in a reverse transcription reaction with SuperScript III (Invitrogen) and random hexamers. Platinum SYBR Green qPCR SuperMix-UDG with ROX (Invitrogen) was used for amplification with a QuantStudio 5 Real-Time PCR system (Thermo Fisher Scientific). Expression values were normalised against actin. Two repeats of four (Islet1) samples at each time point were analysed. qPCR primers used were Islet1 FWD, 5′-TATCAGGTTGTACGGGATCAAA-3′, and REV, 5′-CTACACAGCGGAAACACTCG-3′.

#### Immunohistochemistry and microscopy

Embryos were collected at defined time points and fixed for 30 min for E8.5, 1 h for E9.5 and 2 h for E10.5, in 4% paraformaldehyde in PBS. Embryos for whole-mount imaging were washed in PBS containing 0.1% Triton X-100 (PBST) before the addition of primary antibodies. Embryos for sectioning were placed in cryopreservation with 30% sucrose solution overnight at 4°C, then dissected into forelimb neural tube fragments. These were mounted in gelatine then frozen. Sections (12 μm) were collected on glass slides using a Zeiss Hyrax C 60R cryostat. Gelatine was removed from the slides by 4×5 min washes in PBS at 42°C, and sections were washed with PBST. For *in vitro* stainings, cells were washed in PBS and fixed in 4% paraformaldehyde for 15 min at 4°C, then washed in PBS followed by washing with PBST. For whole embryos, embryo sections and cells, primary antibodies diluted in blocking solution (1% BSA in PBST) were applied overnight at 4°C. These were then washed three times with PBST before secondary antibodies diluted in PBST were added for 1 h at room temperature. Secondary antibodies were removed with three washes with PBST and one wash with PBST and DAPI. Sections and cells were mounted using Prolong Gold (Invitrogen). Embryos for whole mount were mounted using glycerol. Primary antibodies used were as follows: guinea pig anti-Olig2 [a gift from Bennett Novitch, 1:8000 (Novitch et al., 2001)]; mouse anti-Nkx2.2 (BD Pharmingen, 564731, 1:500); rabbit anti-Pax6 (Millipore, AB2237, 1:1000); goat anti-Sox2 (R&D Systems, AF2018, 1:200); mouse anti-Mnx1/HB9 (DSHB, 81.5C10, 1:40); rabbit anti-Olig2 (Millipore, AB9610, 1:1000); goat anti-ISL1 (R&D Systems, AF1837, 1:1000); mouse anti-Chx10 (Santa Cruz Biotechnology, sc-365519, 1:100). All secondary antibodies were raised in donkey and conjugated to Alexa Fluor 488, Alexa Fluor 568 and Alexa Fluor 647 (Abcam). Cells were imaged using a Zeiss Imager Z2 microscope with a 20× objective. The *z*-stacks were taken and presented as a maximum projection using Fiji imaging software. A Leica SP5 upright confocal microscope was used to image embryo sections (40× oil objective) and whole embryos (20× dry objective). For Fig. 2I, images were acquired using a Leica Sp8 inverted confocal microscope (20× dry objective). For whole embryos, *z*-stacks were taken across a tile scan, then assembled and maximally projected using Fiji imaging software (Schindelin et al., 2012).

#### Image quantification

##### Fluorescent intensity measurements

Single optical planes from confocal *z*-stack images were used for analysis. Each nucleus was identified individually using the Fiji point tool. The DAPI channel was used as reference for the position of the nuclei regardless of TF expression. A 2 μm radius circle was taken around each point, *x* and *y* positions, and mean fluorescence intensity values for Nkx2.2, Olig2 and Pax6 were recorded. Reference points at the ventral and dorsal pole of the neural tube in each section were recorded in order to align all embryos along the dorsoventral axis.

##### Pre-processing

We performed a set of normalisation steps in order to compare embryos from different batches and across phenotypes. First, the datasets were realigned vertically with respect to the reference points, and the ventral-most point was set to (0,0) in axes coordinates. Second, cells with DAPI levels below two s.d. from the mean were removed to eliminate falsely identified nuclei. This value was decided individually for each sample to account for different background levels resulting from technical noise. Third, points that were very low in intensity (below two s.d.) were set to a minimum threshold in each individual channel. Fourth, for Nkx2.2 and Olig2, the intensity values were rescaled such that the minimum value was at 0 and the 40% quantile was at the arbitrary value of 0.08. This was carried out individually for each embryo with the assumption that most nuclei in a full neural tube cross-section would not express these proteins. Fifth, for Pax6, most nuclei in the image expressed some level of Pax6; accordingly we set the 60% quantile at 0.6 across all datasets.

##### Staging embryos with size

We used the dorsoventral length of the neural tube as a proxy for embryo age (Cohen et al., 2015). For E9.5 embryos, the neural tube size measured was between 250 μm and 350 μm, and for E10.5 embryos it was larger than 350 μm. In order to subgroup E9.5 embryos, neural tube size was used. In total, we had 46 WT, 29 O2e33^−/−^ and 16 Pax6^−/−^ embryos (Table S1).

##### Classification into cell types

In order to analyse the heterogeneity at the boundary between domains, we classified all cells into one of five specific cell types: floor plate, p3, pMN, Irx3^+^ and other. This was carried out based on the position and expression profile of each cell. We refrained from using the Pax6 channel in our classifier to avoid any bias in the classification of Pax6^−/−^ embryos. We therefore classified on the basis of three parameters: Nkx2.2 intensity, Olig2 intensity and dorsalventral position. The thresholds we employed for Nkx2.2 and Olig2 concentrations are shown in Fig. S21A,B. There was a further constraint on the dorsalventral position for each cell type, in order to avoid anomalies from blood vessels and imaging artefacts, and to be able to separate floor plate cells from Irx3^+^ cells, both of which lack expression of Nkx2.2 and Olig2 (Fig. S21B,C). Manually benchmarking this method indicated that we were able to classify most cells accurately for all three phenotypes. The classifier becomes less accurate for cells in dorsal regions, but this is of no concern as our subsequent analysis did not involve these cells. For the specific task of quantifying the Olig2-Irx3 boundary position, we employed the Pax6 channel as a further parameter to aid classification. This was only performed for WT and O2e33^−/−^.

##### Defining boundary position and width

Once the cell types had been classified, we assigned a quantitative measure of the width of gene expression boundaries. For this, we fitted to the cell position data, for each embryo, a smooth function indicating the probability of finding a cell of one type (the prevalent type on one side of the boundary) at each location of the image. We focused on the boundary between p3 and pMN domains. The classifier is then binary and gives the probability of finding a p3 cell at each image location. We used a Gaussian process approach to fit this classifier as detailed in Rasmussen and Williams (2004), using public MATLAB code (MATLAB version r2018b). The Gaussian process was chosen to have a constant mean function and a squared exponential co-variance function. This choice of co-variance function is relatively standard and allowed us in particular to assign separate co-variance function length scales in the *x* and *y* image directions by automatic relevance determination (Rasmussen and Williams, 2004). We used a logistic transfer function to convert Gaussian process values to probabilities, which was, again, a standard choice. Once the classification probabilities had been obtained in this way, we defined the boundary as the region in which the probability of p3 cells lies in the range of 11% to 89%, i.e. where there is significant mixing of cell types. We then determined the width of this region geometrically. This method allowed us to calculate the boundary widths for all embryos in a consistent manner, and to compare WT with mutants. The boundary region is determined from the trained classifier for each embryo as explained above, and therefore requires no previous knowledge regarding boundary position or width; the position at which the classification probability is 50% for either cell type is used to define the position of the boundary (an average position of the boundary along the left-right axis) (Fig. S22). We did not use entropy based measures, such as those used by Dubuis et al. (2013) and Petkova et al. (2019), as these typically rely on the assumption of Gaussian gene expression level distributions; this assumption is inapplicable in the boundary region, in which the system is bistable and the distributions are therefore bimodal. Information theoretical methods are also normally used with a single spatial coordinate while we are looking at a two-dimensional tissue. This may have irregular growth or oblique sectioning that could lead to a slanted boundary and, therefore, misleading results once projected onto a single dimension.

##### Quantifying TF levels

We extracted Olig2^+^ cells that were classified as being within the boundary region. The model predicted that these cells were the most likely to transition to an Nkx2.2^+^ state given sufficient time. We quantified the levels of Pax6 and Olig2 for these cells in WT and O2e33^−/−^ mutants. The resulting measurements did not provide absolute numbers, but given that all of the samples were normalised in the same way, as described (Materials and Methods), the resulting measurements were comparable relative to each other. We used these measurements as equivalents to observing fluctuations around a steady state over a series of dorsoventral positions. In this way, we took the corresponding equivalent in the simulations, in which we also averaged fluctuations across several neural tube positions (supplementary Materials and Methods).

##### Calculating variance levels

In order to calculate the total variance of Olig2 and Pax6 levels within the pMN domain, we extracted all Olig2-expressing cells, for both WT and O2e33^−/−^, outside the boundary region. The variances and co-variances of the normalised fluorescence intensity values were calculated, in analogy with the theoretical approach (supplementary Materials and Methods). The square root of the trace of the resulting co-variance matrices was then used to obtain the typical root-mean-square relative variance.

## Supplementary Material

Supplementary information
