# Supplementary material for: Precision of tissue patterning is controlled by dynamical properties of gene regulatory networks
Source: Development. 2021 Feb 25;148(4):dev197566. doi: 10.1242/dev.197566 (PMC7929933; doi:10.1242/dev.197566)
Supplement: Supplementary information [file develop-148-197566-s1.pdf]

A Supplementary Figures

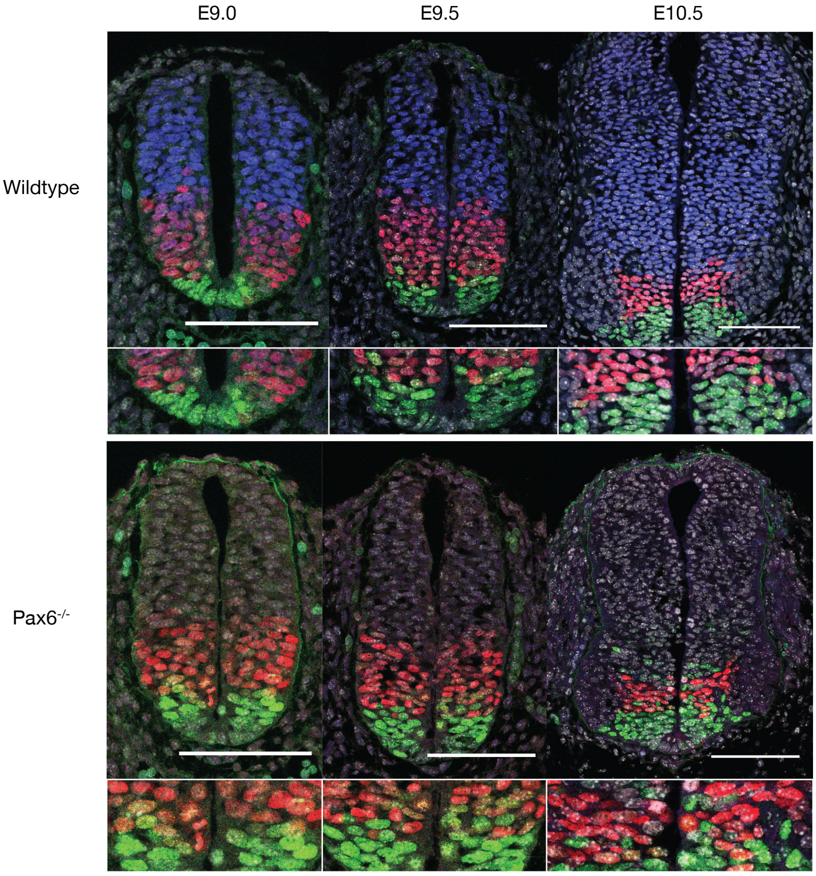

Figure S1: **pMN-p3 boundary precision decreases over time in Pax6 mutants**. Transverse sections of wildtype and Pax6$^{-/-}$ embryos between e9.0 and e10.5 stained for Pax6 (blue), Olig2 (red) and Nkx2.2 (green). Scale bar = 100μm. The pMN-p3 boundary becomes less well defined at later time points.

Development • Supplementary information

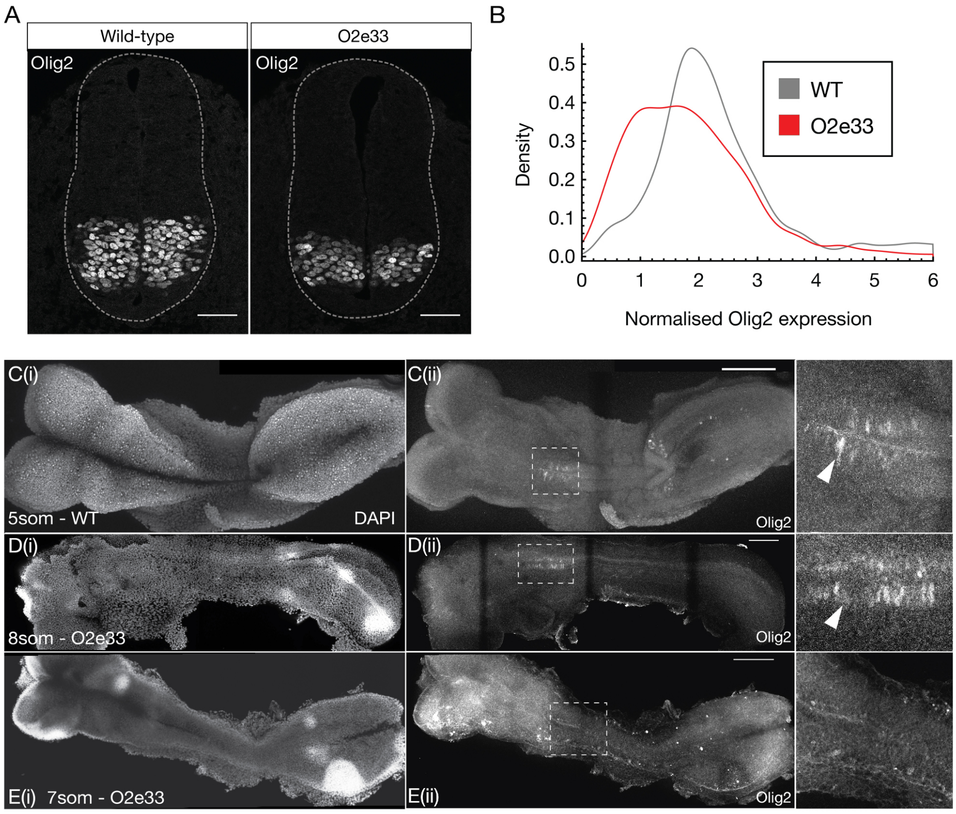

Figure S2: **Olig2 expression in O2e33$^{-/-}$ mutants is lower and delayed in onset**. (**A**) Transverse brachial sections of e9.5 WT and O2e33$^{-/-}$ embryos stained for Olig2. The O2e33$^{-/-}$ embryo has a smaller Olig2 domain with reduced expression levels. Scale bar = 50μm (**B**) Normalised Olig2 expression for single cells in WT and O2e33$^{-/-}$ embryo sections. (**C, D, E**) Wholemount images of WT (C) and O2e33$^{-/-}$ mutants (D, E) for DAPI (i) and Olig2 staining (ii-iii). Expression of Olig2 in wildtype is observed at 5 somites but in O2e33$^{-/-}$ Olig2 onset occurs later at 8 somites. Olig2 is not observed in O2e33$^{-/-}$ embryos at 7 somites. Scale bar = 100μm

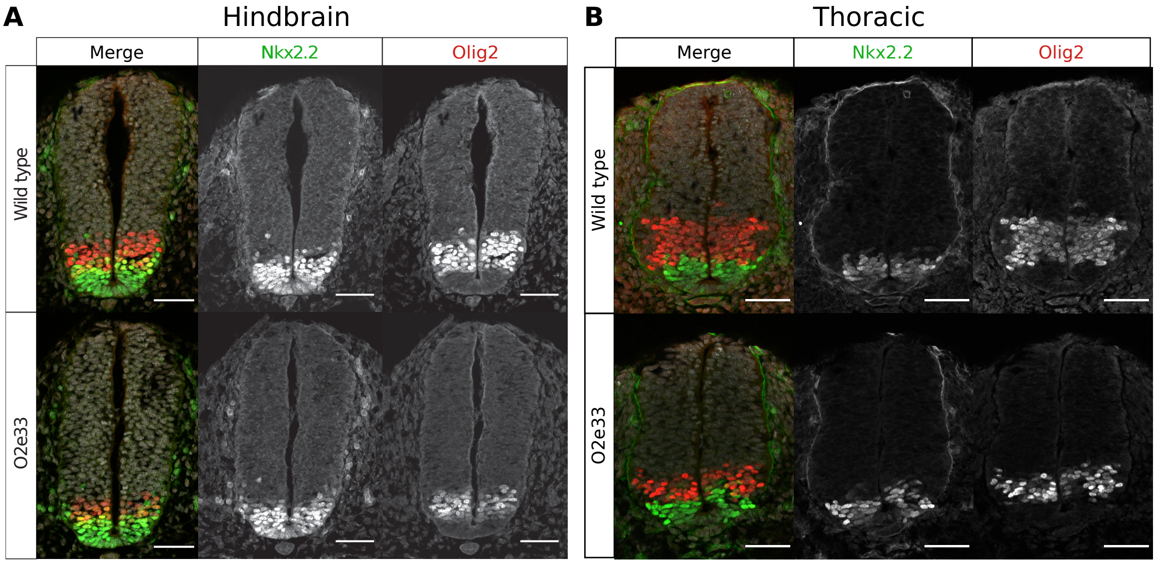

Figure S3: **Transverse sections of the hindbrain (A) and thoracic region (B) e9.5 wildtype and O2e33$^{-/-}$ embryos** Stained for Olig2 (red) and Nkx2.2 (green). Scale bar = 50. (A) Hindbrain: The pMN domain is smaller and the pMN-p3 boundary is less well defined in O2e33$^{-/-}$ mutant embryos. (B) Thoracic region: The pMN domain is smaller and there is more intermixing between pMN and p3 cells in O2e33$^{-/-}$ mutant embryos.

Development • Supplementary information

B Glossary of dynamical systems terminology

The terms in this glossary come from the field of dynamical systems theory and more detail can be found in [Kuznetsov, 2008, Strogatz, 2014]. We then also use elements relating to stochastic processes for which further information can be found in e.g. [Van Kampen, 2007].

- **Deterministic system**

  Deterministic systems are those that involve no randomness and will therefore *always* behave in the exact same way when started from the same conditions. In this study deterministic systems model the production and degradation of genes in the absence of any stochasticity.

- **Stochastic systems**

  These are systems of equations that incorporate randomness such that the system *will not* behave the same way every time. In our study, these are derived from the **deterministic system** by adding a stochastic element to form a **Chemical Langevin Equation**.

- **Chemical Langevin Equation**

  The Langevin equation was derived by Paul Langevin as an equation that approximates the randomness generated by individual processes and has been adapted to describe chemical reaction systems [Lemons and Gythiel, 1997, Gillespie, 2000]. It assumes each individual reaction in a system takes place with Gaussian noise and has been shown to be accurate for systems in which the number of molecules for each component (*e.g.* transcription factor) in the system is sufficiently large. It involves incorporating **stochastic terms**, which describe the noise, into the **deterministic system**.

- **Phase space**

  Phase space is an abstract space in which each dimension represents the concentration of one of the components (transcription factors) of the gene regulatory network. This allows the dynamics of the GRN to be visualised geometrically, such that the change in concentration of the TFs over time traces out a line in phase space.

- **Critical points**

  A critical point is a point in **phase space** where the **deterministic system** does not change over time. That is, the time derivative of all concentrations at a critical point is zero. These points can represent **stable fixed points** of a system, or **unstable fixed points**.

Development • Supplementary information

- **Stable fixed points** (Attractor points)

  A stable fixed point is a type of **critical point**. If in the immediate surroundings of a critical point the dynamics of the **deterministic system** indicate that the system moves towards the fixed point from any direction, this critical point is termed a stable fixed point. This notion is referred to as Lyapunov stability [Lyapunov, 1992]. In a "Waddington-like" landscape visualisation, stable fixed points can be thought of as the basins at the bottom of valleys. In this study, we look at systems with a maximum of two **stable fixed points** (Fig. S4 & S5).

- **Unstable fixed points**

  An unstable fixed point is also a type of **critical point**. In contrast to **stable fixed points**, if the analysis of the **deterministic system** shows that the system moves away from the fixed point when started some small distance away in at least one direction, the point is termed an unstable fixed point. This means that the system will only remain at this point if it is located there *exactly*. A **stochastic system** will not remain at such a point as the stochastic terms will eventually result in the system moving away along an unstable direction. In a "Waddington-like" landscape, unstable fixed points can be thought of as peaks or ridges from which the cell will move away.

- **Saddle point** (Transition point)

  A saddle point is a type of **unstable fixed point** that is attractive in at least one dimension. In the systems within this study (as in many others), saddle points separate **stable fixed points**. A system will approach a saddle point and pass through it during a transition between **stable fixed points**. In a "Waddington-like" landscape, a saddle point appears like a mountain pass between two peaks, or a saddle, hence the name (Fig. S4 & S5).

- **Bifurcation point**

  In the systems in this study bifurcation points are the positions along the morphogen gradient where the system goes from having a single **stable steady state** to being bistable; this means having two **stable steady states** and one **saddle point** (Fig. S4).

- **Fluctuations in concentration**

  In a **stochastic system** the concentrations of the molecules fluctuate *at all times* in a way described by the **Chemical Langevin Equation**. This means that a system never stabilises at a constant concentration, even at a **stable fixed point**. Fluctuations in

Development • Supplementary information

concentration around a stable fixed point remain and can be analysed and visualised in **phase space** (Fig. S5).

- **Noise driven transitions**
  Fluctuations in concentration near a **stable fixed point** move the state of the system away from the **stable fixed point** in phase space. This can result in the system reaching a **saddle point** and as a consequence transitioning from the original **stable fixed point** to the basin of attraction of a different **stable fixed point**. This process is a noise driven transition (Fig. S4 & S5).

- **Minimum Action Path (MAP)**
  From the equations for the **stochastic system** it is possible to calculate the most likely path that a system will take to complete a transition from one **stable fixed point** to another [Kleinert, 2009, Bunin et al., 2012]. This is termed the Minimum Action Path (MAP) and can be visualised as a gene expression trajectory in the **phase space** of TF concentrations. In a "Waddington-like" landscape visualisation such paths can be thought of as the lowest paths in the landscape, which cells are most likely to follow as they move from one state to another(Fig. S5).

In addition, we use the following terms to describe the characteristics of a dynamical system that contribute to precise boundaries.

- **Curvature**
  This is a measurement of how directly the **Minimum Action Path (MAP)** connects a **stable fixed point** to a **saddle point**. In **phase space**, the length of the **MAP** is compared with the shortest distance (straight line) between the initial **stable steady state** and the **saddle point**, at a fixed neural tube position. The greater the ratio between these two distances, the higher the **curvature**.

- **Signal sensitivity**
  A measurement of the Euclidean distance in **phase space** between the **stable fixed point** at which the system starts and the **saddle point**, at a fixed neural tube distance from the **bifurcation point** (the same neural tube position where also the **curvature** is determined). This is termed the signal sensitivity as it indicates how fast the **stable fixed point** and **saddle point** separate in response to distance from the source of morphogen. This term is proposed in the spirit of classical systems theory, where one assesses changes in response to the control parameter, usually with some approximation

Development • Supplementary information

near the bifurcation point. This exact approach is not possible in our case as we are interested in signal sensitivity far from the bifurcation point.

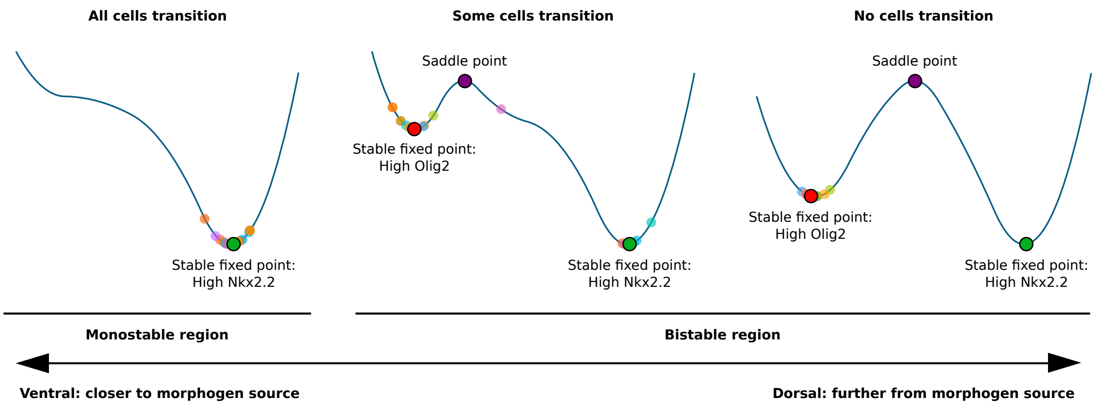

Figure S4: One-dimensional sketches of the dynamical landscape of the neural tube network at multiple dorso-ventral positions as indicated by the bottom arrow. The larger dots with black contours indicate **critical points** as labelled on the plot. The multiple smaller dots represent the final gene expression profile of different simulations of **stochastic** GRN at the same neural tube position. The stochastic nature of the systems leads to cells not following the same identical path. The leftmost plot indicates monostability for high Nkx2.2 near the ventral end of the neural tube; here all cells present high levels of Nkx2.2 as there is no other stable fixed point. In the bistable region all systems start out with high levels of Olig2 as happens in the neural tube. The middle plot represents the landscape slightly dorsal to the **bifurcation point**. The system here presents bistability so that **noise driven transitions** can occur where the system is driven to and beyond the **saddle point**; however these transitions *do not always occur*, leading to heterogeneity in fate decisions for cells at this position. The plot to the right represents cells much further dorsal of the bifurcation; here the probability of a system reaching the **saddle point** is extremely low even with stochastic terms as can be appreciated from the figure. In this region despite the existence of bistability, only the high Olig2 **stable fixed point** is observed as the probability for **noise driven transitions** to occur is vanishingly small.

Development • Supplementary information

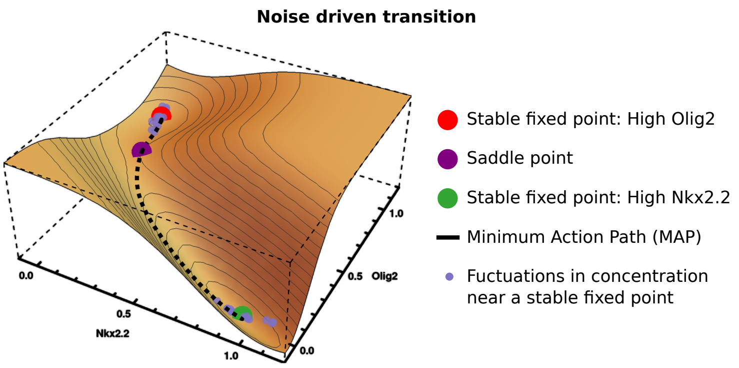

Figure S5: Sketch of an approximate dynamical landscape for the neural tube network at a region of bistability, slightly dorsal to the **bifurcation point**. The x and y axes are the concentrations of Nkx2.2 and Olig2 respectively (2D phase space), whereas the z axis represents the landscape of the system. The plot relates to the same neural tube position as the middle figure in Fig. S4, therefore there is heterogeneity in fate decisions. The colouring of the **critical points** is consistent with Fig. S4; see also the legend. The light blue dots represent two different simulations near each **stable fixed point**, illustrating **fluctuations in concentration**. The thick black line illustrates the **MAP** from the high Olig2 **stable fixed point** to the high Nkx2.2 **stable fixed point**. Note that it passes through a critical point – a saddle (purple dot). Typical transitions from one stable fixed point to another are one-dimensional in the sense that they proceed along the MAP; we can define an effective landsape.

Development • Supplementary information

C Formulation and analysis of stochastic GRN dynamics

Formulation of stochastic dynamics

In order to investigate heterogeneity of gene expression in the neural tube we made use of stochastic differential equations that describe the GRN and in particular the time evolution of the concentration x_j of each TF j. We start with a thermodynamic-like model as detailed in [Cohen et al., 2014], which captures the macroscopic behaviour by a system of ODEs; these contain terms for production and decay of each TF. The ODE description corresponds to the limit of a reaction volume Ω that is large enough for the copy numbers Ωx_j of all protein species to be large, allowing fluctuations to be neglected; formally one takes $\Omega \to \infty$. When Ω is finite, stochastic effects occur. These can be described by the chemical Langevin equation, a system of SDEs, see e.g. [Van Kampen, 2007, Gillespie, 2000]. The drift, *i.e.* the systematic variation with time in the SDEs coincides directly with the deterministic limit. The diffusion (stochastic) term arises from the stochastic nature of the individual protein production and decay reactions; it is a Gaussian white noise [Gillespie, 2000] whose covariance structure is determined by the mean reaction rates. In our case the chemical Langevin equation for the protein levels x_j within the GRN takes the form:

$$\frac{d}{dt}x_j = \sum_{\boldsymbol{n}} p_{(j,\boldsymbol{n})}\alpha_{(j,\boldsymbol{n})} - x_j\beta_j + \Omega^{-1/2}\epsilon_j(t) \tag{C.1a}$$

$$p_{(j,\boldsymbol{n})} = \frac{k_{(j,n)}\prod_i x_i^{n_i}}{\sum_{n'} k_{(j,n')}\prod_i x_i^{n'_i}}$$

$$D_{ij} = \delta_{ij}\left[\sum_{\boldsymbol{n}} p_{(j,\boldsymbol{n})}\alpha_{(j,\boldsymbol{n})} + x_j\beta_j\right] \tag{C.1b}$$

$$\langle \epsilon_i(t)\epsilon_j(t')\rangle = \delta(t-t')D_{ij} \tag{C.1c}$$

The deterministic part of these equations is equivalent to those used in [Cohen et al., 2014]. The covariance (C.1b,C.1c) of the zero mean Gaussian white noise $\epsilon_j(t)$ arises from the decay and production of each protein being independent and random, given the concentration of the regulators of the relevant gene. In the equations above, α represents protein production rate and β degradation rate, while the w provide the weights of the respective DNA conformations $(j, \boldsymbol{n})$ when multiplied by the respective concentration. The conformations are labelled by the protein j being produced and the numbers $\boldsymbol{n} = \{n_i\}$ of TF molecules bound. The δ in (C.1b) and (C.1c) are the Kronecker and Dirac delta respectively. As explained above, Ω is the volume of the system in which all reactions take place.

When looking at the chemical Langevin equation (C.1a), one notices that the rate $\sum_{\boldsymbol{n}} p_{(j,\boldsymbol{n})}\alpha_{(j,\boldsymbol{n})}$ for producing protein j, has a nonlinear dependence on the TF concentrations x_i. One might be concerned that with such a nonlinear dependence, modelling production of protein j as a single reaction is too simplistic. However, (C.1a) can be obtained from a larger system of simple unary and binary mass action reactions, in which the concentration of each DNA conformation is kept track of individually. We only sketch this construction here and explain its implications for the stochastic terms in (C.1a); for further details see [Herrera-Delgado et al., 2018]. The deterministic part of the time evolution of the DNA concentrations is given as follows:

$$\frac{d}{dt}x_{(j,\boldsymbol{n})} = \gamma \sum_p \left(k^{p+}_{(j,\boldsymbol{n}-\boldsymbol{e}_p)}x_{(j,\boldsymbol{n}-\boldsymbol{e}_p)}x_p - k^{p+}_{(j,\boldsymbol{n})}x_{(j,\boldsymbol{n})}x_p + k^{p-}_{(j,\boldsymbol{n}+\boldsymbol{e}_p)}x_{(j,\boldsymbol{n}+\boldsymbol{e}_p)} - k^{p-}_{(j,\boldsymbol{n})}x_{(j,\boldsymbol{n})}\right) \tag{C.2}$$

Here $x_{(j,\boldsymbol{n})} = \tilde{x}_{(j,\boldsymbol{n})}/\gamma'$ tracks the concentration of each DNA conformation and is scaled down by a large factor γ' to account for the low quantity of binding sites in relation to protein numbers. Correspondingly the protein production rate constants $\alpha_{(j,\boldsymbol{n})} = \gamma'\tilde{\alpha}_{(j,\boldsymbol{n})}$ have to be large in order to give an appreciable overall rate of protein production nonetheless.

To derive the correct stochastic equations for the protein species, the large γ-limit of (C.2) is taken: the concentration of each DNA conformation then changes sufficiently quickly that it constantly tracks the instantaneous protein concentrations. For appropriately chosen binding and unbinding rate constants $k^{p+}_{(j,\boldsymbol{n})}$ and $k^{p-}_{(j,\boldsymbol{n})}$ this leads back to the thermodynamic-like form of the *deterministic* part of the protein dynamics in (C.1a) [Herrera-Delgado et al., 2018]. As shown in [Thomas et al., 2012] the existence of fast species (in our case, DNA conformations) can lead to additional terms arising in the *noise* acting on the slow species (protein production), as a consequence of reactions between slow and fast species. In our case it turns out that these extra noise terms scale with γ'/γ. We then make use of the biological meaning of the terms: $1/\gamma$ represents the timescale of reaction rates for TF binding to DNA and $1/\gamma'$ represents the characteristic time for the process of going from active DNA to producing a protein. We find it biologically reasonable to choose a $1/\gamma$ that is substantially smaller than $1/\gamma'$, given the many biological processes necessary for the production of a fully functional protein. The ratio γ'/γ is then small so that the additional noise terms that arise from the general calculation in [Thomas et al., 2012] become negligible, leaving exactly the noise terms in (C.1c). The intuition is that because protein production is slow compared to binding and unbinding of factors to DNA, noise from the many binding and unbinding events during production averages out; the overall noise then arises only from the stochasticity of the production processes, at the relevant average DNA concentrations. We note that in accordance with this conclusion, explicit calculations

Development • Supplementary information

show that when γ' is of the order of γ or larger, additional noise terms from the stochasticity in DNA concentrations do enter the dynamics of the protein concentrations. Moreover, these additional terms are dependent on the precise choices of binding and unbinding rates, which are only partially constrained by the requirement that the thermodynamic-like deterministic equations (C.1a) are retrieved for large γ [Herrera-Delgado et al., 2018].

Amount of noise

The noise level in our model is set by Ω^{-1}, the inverse reaction volume. This determines the scale of the stochastic fluctuations in protein production and decay, both of which the model represents as single step processes. A larger Ω thus leads to smaller stochastic effects. In equation (C.1a), multiplying Ω by the concentration of a protein species gives the number of molecules for that protein. In our calculations we measure volumes in units that make typical protein concentrations of order unity, so that Ω can be interpreted as a copy number. In accordance with our observations in (Supp. D), a value for Ω can be read as a copy number for Pax6, Nkx2.2 and Irx3; the corresponding typical copy numbers for Olig2 are ten times higher (Supp. D).

However, the model is a coarse-grained description that does not explicitly describe the many possible sources of noise within a living cell. These include spatial heterogeneity and effects from the bursty, multi-step nature of protein production, which includes processes such as transcription, translation, post-translational modification, protein folding and protein shuttling [McAdams and Arkin, 1997]. As noted in [Van Kampen, 2007] and as implemented in [Wang et al., 2007, Zhang et al., 2012, Li and Wang, 2013], Ω relates inversely to the magnitude of fluctuations at a macroscale. It therefore represents the combined effect of all the processes involved in gene regulation and protein production that contribute to the overall system noise. Hence Ω is an “effective” system size parameter, which incorporates all the stochastic effects in the system. Of particular relevance, mRNA molecule number is typically one thousandth that of protein number [Schwanhäusser et al., 2011]. Consistent with this we have found an average of ~100,000 Olig2 protein molecules/cell but only ~40 Olig2 mRNA molecules/cell [Rayon et al., 2019].

We therefore set out to estimate lower and upper bounds on the noise level Ω^{-1}, *i.e.* the range of noise that makes sense within our description. The lower bound is given by the typical number of proteins of each species in a cell: these numbers determine the minimum amount of noise that must arise from the stochastic nature of protein production and decay. From protein quantifications (Supp. D) we obtain $\Omega_{\max} \sim 10,000$ for the protein counts of Nkx2.2

Development • Supplementary information

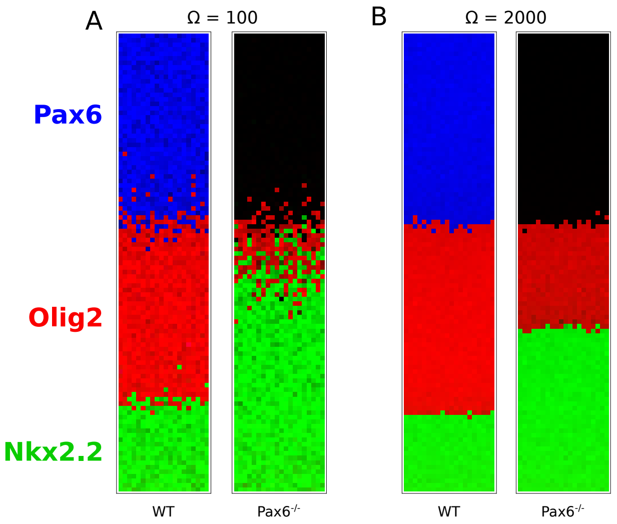

Figure S6: **Simulations of the WT and Pax6$^{-/-}$ stochastic models** for (A) $= 100$, (B) $\Omega = 2000$. For this range for Ω the simulations recapitulate the observed relationship of boundary sharpness and position in WT and Pax6$^{-/-}$ mutants.

and Pax6 per cell at saturation levels (which in our model correspond to concentrations close to unity). Olig2 has a higher estimated count of ~100,000 and in accordance a 10 times higher concentration in the model (the maximum concentration for Olig2 is 10, and 1 for the other TFs). Because of the many neglected sources of additional noise, we expect $1/\Omega_{\max}$ to be a considerable underestimate; indeed, simulations with this noise level show almost deterministic behaviour. However, already for a slightly increased noise level ($\Omega = 2000$), we find that the relationships between jump-rate differences across WT and mutant phenotypes discussed in the main text hold true (see Fig. S6). In particular, the WT presents a small amount of heterogeneity (as observed *in vivo*) and the mutants have a more heterogeneous boundary than the WT.

To obtain a lower bound for Ω, we measured the coefficient of variation at steady state for all 3 TF values across embryos, to estimate the total amount of noise in the system (Fig. 1A). We then decrease Ω in our numerical simulations until we see coefficients of variation similar to those observed *in vivo*, giving $\Omega_{\min} = 20$. This assumes that *all* observed differences in protein levels arise *solely* from the stochasticity in our model. We reason that there are other sources of noise that make the coefficients of variation higher *in vivo*, such as noise resulting from transcription, protein transport within the cell, antibody specificity and measurement error, so that the amount of noise contributed by the stochasticity in our dynamical model will be smaller than $1/\Omega_{\min} = 1/20$. On that basis we find a reasonable smallest value of Ω of ~ 100. The value we use for all results throughout this study is $\Omega = 250$, which is within the broad bounds of $\Omega_{\min} = 20$ and $\Omega_{\max} = 20,000$. Importantly, the results we observe

Development • Supplementary information

remain qualitatively unchanged across the entire range of Ω that we assess as reasonable, $100 \leq \Omega \leq 2000$ (Fig. S6).

To confirm that the effective Ω provides a reasonable estimate of the effect of noise, we performed simulations of the GRN that incorporate the mRNA as well as the protein steps for the production of TFs as additional variables in the system. For this we use experimentally determined mRNA levels [Rayon et al., 2019]. With this addition, protein levels of between 10^4 and 10^5 molecules/cell and mRNA levels of $\sim$50 molecules/cell recapitulate the experimentally observed variance in protein levels and the stochasticity of cell fate transitions (Fig. S7). Since it is not our aim to add unnecessary complexity to the model, we did not include mRNA steps in our further analyses.

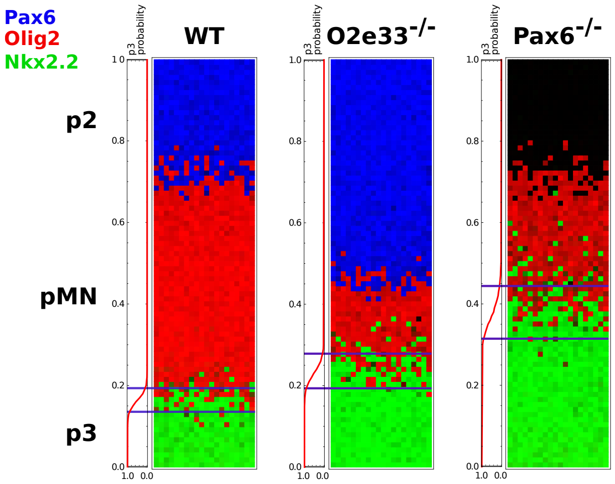

Figure S7: Stochastic simulations of the three genotypes incorporating mRNA as well as protein production. Setup consistent with the simulations shown in the manuscript, with the same colour code and top-down being dorso-ventral. The simulated model includes transcription and translation by including mRNA and protein concentrations for each TF. The simulations use a copy number of 100,000 protein/molecules per cell for Olig2 and 10,000 for the other TFs. For mRNA of Olig2, 40 molecules per cell were used, and 20 molecules for all other mRNA numbers. Parameters for transcription and translation have been extracted from experimental measurements [Rayon et al., 2019]. The lines along each simulation graph show the probability of finding a cell in the p3 state at each neural tube position, with the lines indicating the boundary width extracted from these probabilities.

Minimum action path

Much of the theoretical analysis in the main text concentrates on the stochastic transitions between fixed points of the deterministic GRN dynamics, which are long-lived metastable states of the stochastic dynamics. The minimum action path (MAP) is the most likely path the system takes in such a transition (for large enough values of Ω), from a steady state to

Development • Supplementary information

a transition point (which is the saddle point of the dynamical system) and then onwards to a new steady state. The second piece of the path always follows the deterministic dynamics and has a negligible effect on the transition times, so we focus on the first part of the path.

The negative log probability for any path is proportional to what is called the action, which for our Langevin dynamics is of so-called Onsager-Machlup form [Kleinert, 2009]. The action is an integral over time of the Lagrangian, which in turn depends only on the current state (vector of concentrations) and velocity of the system. The time integral can be discretised and the action then minimised as described in e.g. [Bunin et al., 2012]. We analyse the resulting MAP in gene expression space in order to understand how its shape affects the jump times between steady states and thus eventually the boundary width.

The typical time the system takes to reach any point on the MAP scales exponentially with the action up to that point, hence this quantity can be interpreted as an effective energy, within the analogy of a particle making a transition from one local minimum in an energy landscape across a barrier to another minimum. In Fig. 4F we plot this effective energy along the (relative) length of the MAP, describing the effective energy landscape governing the transition. Fig. S8 shows an alternative representation that gives further insight: we plot the derivative of the action along the path, which is the effective force pushing the system back towards the initial steady state.

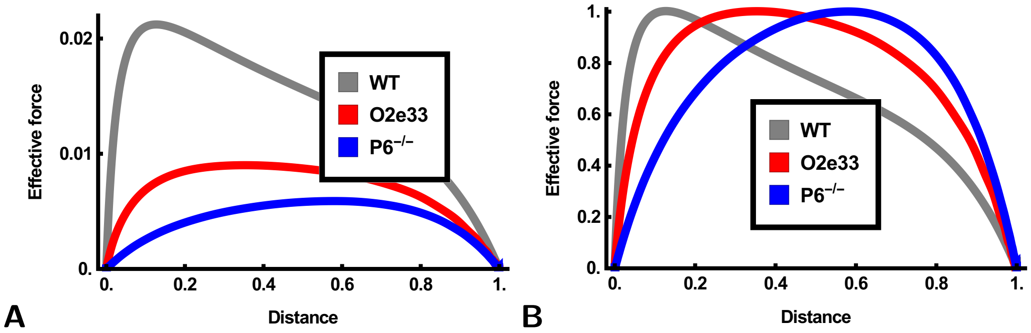

Figure S8: (**A**-**B**) Unnormalised and normalised space derivative of the action along the MAP, plotted along the length of the path. This reflects the effective force driving the system back towards its initial steady state. In the WT system (gray) the force is highest near the beginning of the path, leading to a noticeably skewed plot, while the O2e33$^{-/-}$ (red) and Pax6$^{-/-}$ (blue) more nearly symmetric force profiles. The high initial force in WT responsible for the large typical jump times in the system, and is related to the significant curvature of the MAP away from the straight line between initial steady state and transition point (Fig. 4D-F)

Calculating magnitude of fluctuations

To compare the magnitude of fluctuations between WT and mutants *in silico* we take two separate approaches. The first is to consider fluctuations in expression levels around a steady state, before any transition to a new state occurs. For moderate noise levels such fluctuations can be analysed using a linear expansion of the dynamics around the steady state (here: pMN), leading to a local Gaussian distribution of expression levels. The corresponding covariance matrix $\boldsymbol{C}$ can be calculated from the Jacobian matrix $\boldsymbol{J}$ of the linearized dynamics and the noise covariance $\boldsymbol{D}$ as defined in (C.1b), both evaluated at the steady state. The required link between the three matrices is the Lyapunov equation, which determines $\boldsymbol{C}$ via

$$\boldsymbol{D} = \boldsymbol{JC} + \boldsymbol{CJ}^{\mathrm{T}}$$

Once $\boldsymbol{C}$ has been found we normalise it by the corresponding pMN steady state values ($\boldsymbol{X}$), to obtain $\bar{\boldsymbol{C}} = \mathrm{diag}(\boldsymbol{X})^{-1}\boldsymbol{C}\mathrm{diag}(\boldsymbol{X})^{-1}$. We finally compute the trace of $\bar{\boldsymbol{C}}$ and take the square root. The end result is the typical standard deviation (root-mean-square fluctuation) of the expression levels, relative to the mean expression levels. This is shown in Fig. S9**A** as a function of neural tube position.

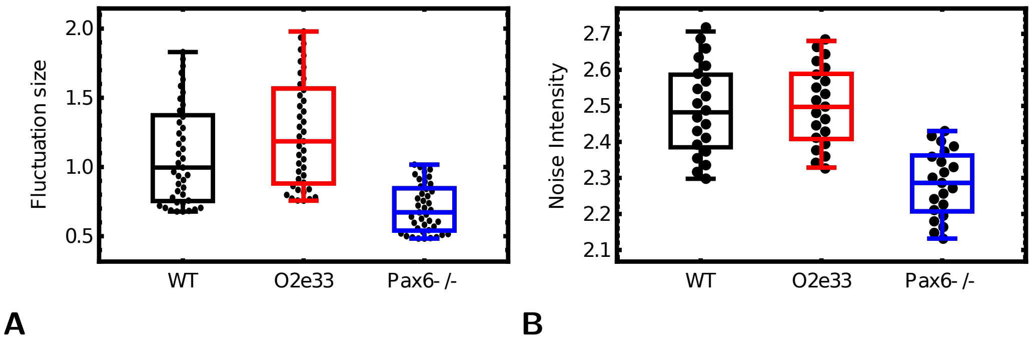

Figure S9: **Comparing total noise across genotypes** (A) Comparison of noise levels as defined by root-mean-square relative expression level fluctuations, calculated within a Gaussian approximation near the steady state. Points represent different positions along the neural tube (B) Noise levels defined as noise variance calculated at equidistant points along the MAP, at fixed fractional neural tube length from the bifurcation point. Note that in both definitions, noise levels are comparable across WT and both mutants, with slightly lower values in Pax6$^{-/-}$.

The second approach to quantifying noise levels is to use the noise variance, which is the trace of the noise covariance matrix given in (C.1b). This noise variance depends on the expression levels so we average it across equidistant points along the MAP and take the square root of this value to obtain the root-mean-square noise level. Example results at a specific position along the neural tube are shown in Fig. S9B; results at other positions

Development • Supplementary information

were qualitatively the same (data not shown). Both approaches to quantifying noise show comparable total variance across the different genotypes, with slightly lower noise in Pax6$^{-/-}$ than in WT and O2e33$^{-/-}$. To make the comparison to *in vivo* observations we accounted for the fact that experimentally, noise levels are averaged across several neural tube positions throughout the pMN domain. We therefore also performed an average *in silico* of neural tube positions to obtain comparable data for Fig. 3**D**.

D Protein Number Quantifications

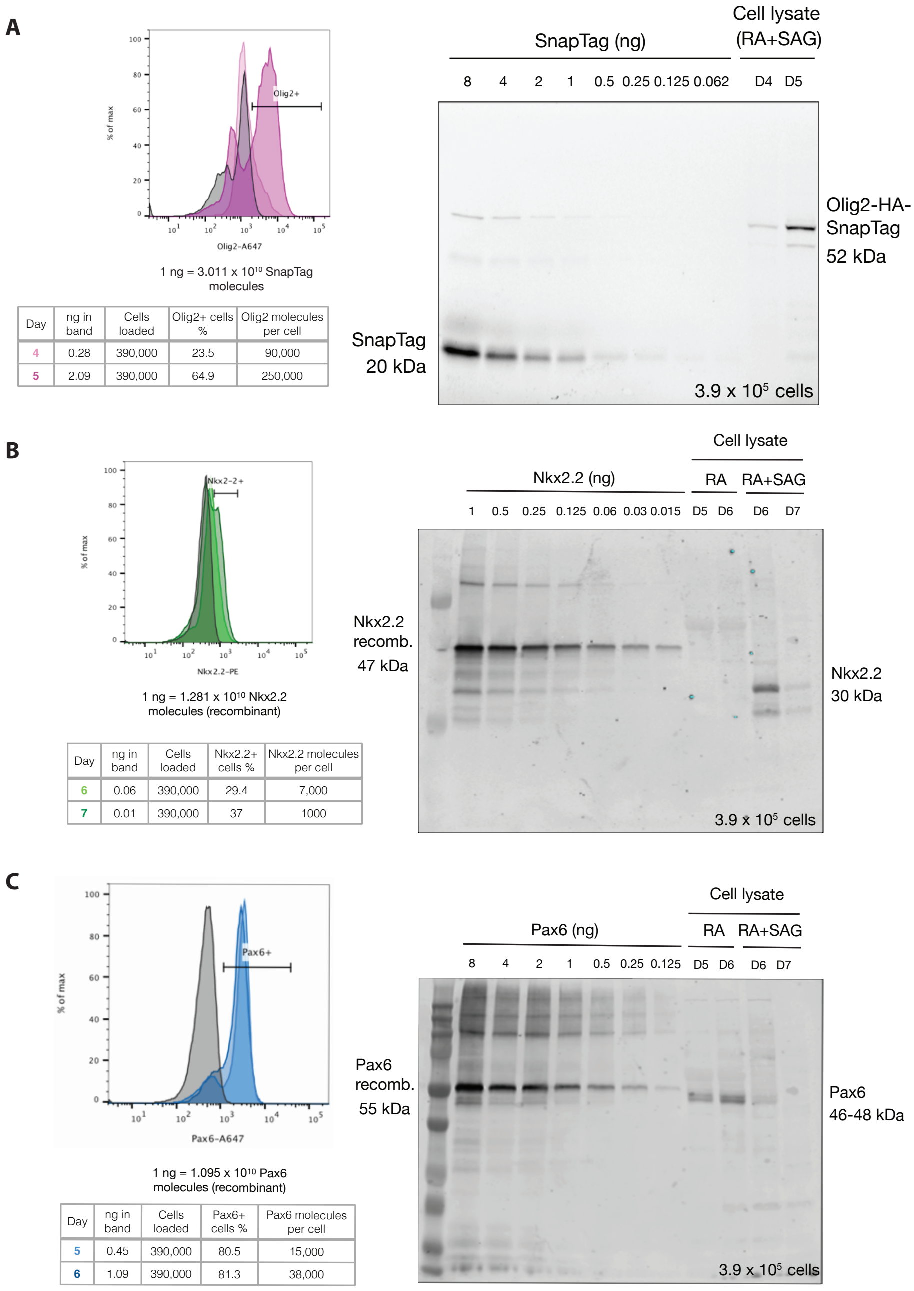

Day	ng in band	Cells loaded	Olig2+ cells %	Olig2 molecules per cell
4	0.28	390,000	23.5	90,000
5	2.09	390,000	64.9	250,000

Day	ng in band	Cells loaded	Nkx2.2+ cells %	Nkx2.2 molecules per cell
6	0.06	390,000	29.4	7,000
7	0.01	390,000	37	1000

Day	ng in band	Cells loaded	Pax6+ cells %	Pax6 molecules per cell
5	0.45	390,000	80.5	15,000
6	1.09	390,000	81.3	38,000

Figure S10: **Quantifying Protein Copy Number** (**A**) Flow cytometry analysis to determine percentage of Olig2 expressing cells in differentiated ES cells at the indicated days. Table shows quantification of a gel for days 4 and 5. Olig2 has approximately a 10-fold higher protein copy number compared to Nkx2.2 and Pax6. (**B**) Analysis of Nkx2.2 expressing cells on days 6 and 7 of differentiation. Nkx2.2 molecules per cell calculated using the measured percentage of cells expressing Nkx2.2 and quantification of the Western blot analysis. (**C**) Analysis of Pax6 expressing cells to determine protein copy number at days 5 and 6 of differentiation. Pax6 molecules per cell calculated using the measured percentage of cells expressing Pax6 and quantification of the Western blot analysis.

Development • Supplementary information

E Simulating WT and mutant GRNs

We used the equations and parameters described in [Cohen et al., 2014] for the GRN that patterns the neural tube; this parameter set was optimised to replicate the boundary positions in wild-type and mutant embryos. Following the inclusion of the noise term as explained in Supp. C we explored the effect of the initial conditions for the TFs (i.e. their initial expression levels x_j). The aim was to find a consistent set of initial conditions that sustain the boundary positions but also recapitulate the boundary sharpness of each mutant. The initial conditions that satisfied these conditions were identified in a systematic scan as $x_{\mathrm{Pax6}} = 0.1$, $x_{\mathrm{Olig2}} = 0$, $x_{\mathrm{Nkx2.2}} = 0$, $x_{\mathrm{Irx3}} = 0.1$. The p3-pMN boundaries in WT, Irx3$^{-/-}$, Nkx2.2$^{-/-}$ and Olig2$^{-/-}$ simulations remained sharp as is the case *in vivo* (Fig. S11). Only the loss of Pax6 resulted in decreased boundary sharpness. Boundary positions remained consistent with *in vivo* observations as was the case in the original deterministic model (Fig. S11) & [Cohen et al., 2014].

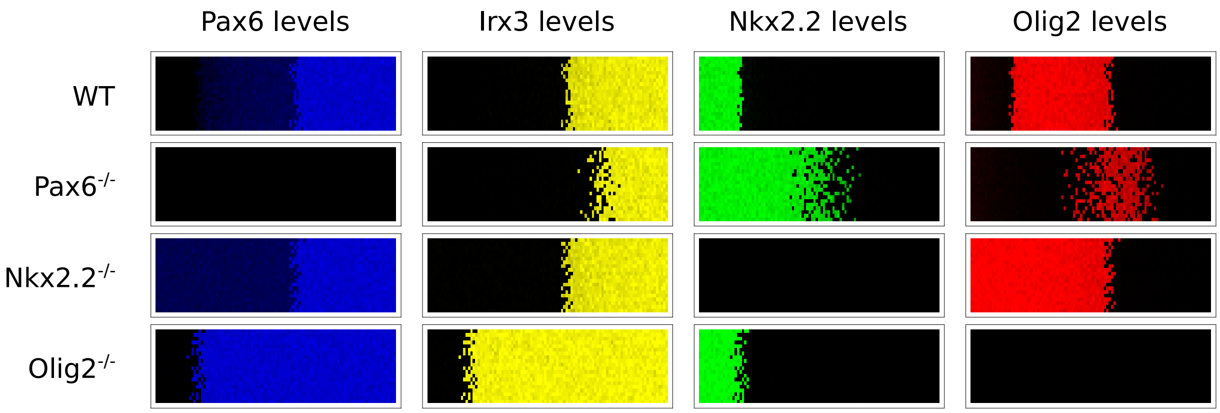

Figure S11: **Patterning phenotypes produced by stochastic simulations for WT and mutants**. Predicted expression patterns for the four TFs in the indicated genotypes are qualitatively similar to those in [Cohen et al., 2014]. Ventral to the left and dorsal to the right. Although boundary positions change, boundary precision is largely unaffected except for Pax6$^{-/-}$, consistent with *in vivo* experimental observations.

Model parameters

We detail the parameters used throughout the paper to model neural tube development for equation (C.1a), and adapted for the computational screen as explained in Supp. F.

Development • Supplementary information

Name	Meaning	Value	Source
α_{P}	Pax6 production rate	2	[Cohen et al., 2014]
α_{O}	Olig2 production rate	2×10	[Cohen et al., 2014] & Supp. C
α_{N}	Nkx2.2 production rate	2	[Cohen et al., 2014]
α_{I}	Irx3 production rate	2	[Cohen et al., 2014]
β_{P}	Pax6 degradation rate	2	[Cohen et al., 2014]
β_{O}	Olig2 degradation rate	2	[Cohen et al., 2014]
β_{N}	Nkx2.2 degradation rate	2	[Cohen et al., 2014]
β_{I}	Irx3 degradation rate	2	[Cohen et al., 2014]
k_{PO}	Olig2 binding to Pax6 DNA	1.9/10	[Cohen et al., 2014] & Supp. C
k_{PN}	Nkx2.2 binding to Pax6 DNA	26.7	[Cohen et al., 2014]
k_{ON}	Nkx2.2 binding to Olig2 DNA	60.6	[Cohen et al., 2014]
k_{OI}	Irx3 binding to Olig2 DNA	28.4	[Cohen et al., 2014]
k_{NP}	Pax6 binding to Nkx2.2 DNA	4.8	[Cohen et al., 2014]
k_{NO}	Olig2 binding to Nkx2.2 DNA	27.1/10	[Cohen et al., 2014] & Supp. C
k_{NI}	Irx3 binding to Nkx2.2 DNA	47.1	[Cohen et al., 2014]
k_{IO}	Olig2 binding to Irx3 DNA	58.8/10	[Cohen et al., 2014] & Supp. C
k_{IN}	Nkx2.2 binding to Irx3 DNA	76.2	[Cohen et al., 2014]
$w_{\mathrm{P,p}}$	Polymerase binding to Pax6 DNA	3.84	[Cohen et al., 2014]
$w_{\mathrm{O,p}}$	Polymerase binding to Olig2 DNA	2.01263	Converted from [Cohen et al., 2014]
$w_{\mathrm{N,p}}$	Polymerase binding to Nkx2.2 DNA	0.572324	Converted from [Cohen et al., 2014]
$w_{\mathrm{I,p}}$	Polymerase binding to Irx3 DNA	18.72	[Cohen et al., 2014]
$k_{\mathrm{O,in}}$	Gli (Shh signal) binding to Olig2 DNA	180	Converted from [Cohen et al., 2014]
$k_{\mathrm{N,in}}$	Gli (Shh signal) binding to Nkx2.2 DNA	373	Converted from [Cohen et al., 2014]
Ω	System volume	250	Supp. C
$x_{\mathrm{P}}(0)$	Pax6 initial condition	0.1	Supp. E
$x_{\mathrm{O}}(0)$	Olig2 initial condition	0	Supp. E
$x_{\mathrm{N}}(0)$	Nkx2.2 initial condition	0	Supp. E
$x_{\mathrm{I}}(0)$	Irx3 initial condition	0.1	Supp. E

Where factors of 10 have been written in the table, these arise because we have modified the model of [Cohen et al., 2014] to represent explicitly the experimental observation that Olig2 has a concentration 10 times higher than the other TFs. While this difference is immaterial for a deterministic description of the GRN dynamics, it affects the stochastic representation because larger copy numbers have smaller relative fluctuations.

The above parameters are used in the general model (C.1a) for the dynamics of the TFs j = P (Pax6), O (Olig2), N (Nkx2.2) and I (Irx3). DNA conformations are defined by the numbers $\boldsymbol{n} = (n_{\mathrm{p}}, n_{\mathrm{in}}, n_{\mathrm{P}}, n_{\mathrm{O}}, n_{\mathrm{N}}, n_{\mathrm{I}})$ of bound molecules of polymerase, Gli signal input, Pax6, Olig2, Nkx2.2, Irx3 in that order. The only allowed conformations are the empty conformation, the conformations with polymerase and $n_{\mathrm{in}} = 0$ or 1 signal molecule bound; and conformations with at least one molecule of the other TFs bound, with maximally two molecules from each other TF. All other conformations are assigned affinity zero. The weights

Development • Supplementary information

for the allowed conformations are multiplicative, with bound polymerase contributing a factor $w_{j,\mathrm{p}}$ (see below), bound signal a factor $k_{j,\mathrm{in}}x_{\mathrm{in}}$ and each TF i bound to DNA producing TF j a factor $k_{ji}x_i$. Examples of the corresponding affinities are $k_{\mathrm{O},(0,0,0,0,1,0)} = k_{\mathrm{ON}}$ and $k_{\mathrm{O},(0,0,0,0,0,2)} = k_{\mathrm{OI}}{}^2$. The polymerase binding parameters are directly stated as the weights $w_{j,\mathrm{p}} = k_{j,\mathrm{p}}x_{\mathrm{p}}$ including polymerase concentration (which is assumed constant). As detailed in [Cohen et al., 2014], this weight describes all basal production inputs for each TF and thus represents input from TFs such as Sox2 [Graham et al., 2003, Oosterveen et al., 2012, Peterson et al., 2012]. Finally, the protein production rates $\alpha_{j,\boldsymbol{n}}$ in the general model (C.1a) are set to the value given in the table for the DNA conformations with bound polymerase, and zero otherwise.

As an explicit example of the resulting GRN equations, we write here the production rate for Olig2:

$$\frac{\alpha_{\mathrm{O}} w_{\mathrm{O,p}}(1 + k_{\mathrm{O,in}}x_{\mathrm{in}})}{w_{\mathrm{O,p}}(1 + k_{\mathrm{O,in}}x_{\mathrm{in}}) + (1 + k_{\mathrm{OI}}x_{\mathrm{I}})^2(1 + k_{\mathrm{ON}}x_{\mathrm{N}})^2} \tag{E.1}$$

The signal input concentration x_{in} is the gradient $e^{-s/0.15}$, which depends on the dorsal-ventral neural tube position $s$ranging from 0 to 1 as in [Cohen et al., 2014].

Development • Supplementary information

O2e33$^{-/-}$ mutant

To find parameter sets that describe the behaviour of the O2e33$^{-/-}$ enhancer mutation, we first identified those parameters that are related directly to the deletion of the respective enhancer. Analysis of the sequence of the enhancer together with CHIP-seq and ATAC-seq [Oosterveen et al., 2012, Peterson et al., 2012, Kutejova et al., 2016, Metzis et al., 2018] suggested that Gli proteins, Nkx2.2, Irx3, and Sox2 all have a direct effect on this enhancer (Fig. 2A). We therefore considered variations in the parameters that specify Nkx2.2 binding, Irx3 binding, Gli binding and basal production (corresponding to Sox2 binding). We systematically explored how reducing the parameters for each of these interactions, to a fraction f of their original value, could explain the observed phenotype. We used a uniform distribution to perform this search and throughout this supplementary represent the respective parameter reductions directly in terms of the ratio f between new and original (WT) parameter values.

Fitting *in vitro* delay and resulting predictions

We first identified parameter sets that could replicate the observed *in vitro* delay in the onset of Olig2 expression in the mutant, leading to a reduced parameter space (Fig. S12). In this step we do not set any constraints to the position or precision of boundaries between expression domains as this information cannot be extracted from the *in vitro* system. The delay in Olig2 activation was determined for networks positioned a fraction 0.3 along the neural tube, and we retained those networks that took twice the amount of time to express Olig2 than in the WT. The same measurement was performed at other neural tube positions and resulted in similar distributions (data not shown).

We next investigated what further phenotypical behaviour the retained parameter sets predict, focussing on the domain size and boundary precision generated in response to a graded Shh signal. We found that 68% of the parameter combinations reduced boundary precision, 80% reduced the size of the pMN domain, with 83% presenting one or the other of the phenotypes (data not shown). Here, the pMN domain size was calculated with respect to the Shh gradient and we considered it reduced if it was below 70% of the WT size. For determining boundary sharpness, we regarded as imprecise those systems that had a boundary width at least twice the size of the WT; this width is calculated using the SDE system with the thresholds described in Fig. 3B. The fact that a majority of the parameter sets identified affected domain size and boundary precision encouraged us to generate the mouse lines.

Development • Supplementary information

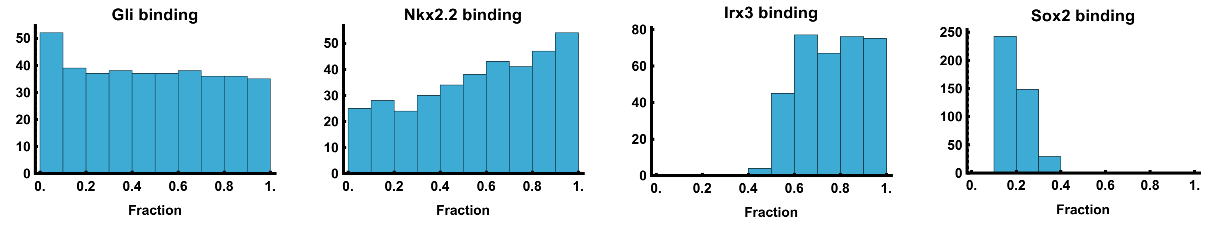

Figure S12: **Distribution of parameter changes to mimic *in vitro* O2e33$^{-/-}$ mutant.** To recapitulate the O2e33$^{-/-}$ dynamics *in vitro*, model parameters were systematically explored to identify changes that could account for the delay in onset of Olig2 expression. The graphs show the distributions of reduction factors f (x-axis) relative to WT parameter values, across parameter sets that recapitulate the delay. The (y-axis) shows number of parameter combinations that recapitulate the phenotype. The results show that what is needed to generate a delayed induction of Olig2 is a substantial reduction in Sox2 input while maintaining input of Irx3.

Fitting *in vivo* phenotype with patterning information

Once the mouse lines were generated we confirmed the delay in onset of Olig2, and noted two additional phenotypes as expected from the initial parameter screen: a loss of precision at the p3-pMN boundary and a ventral shift of the pMN-p2 boundary. Importantly, this *in vivo* data allowed us to define targets regarding boundary position and precision for our fitting of the mutant phenotype. The new targets were therefore extracted from this data, and were used to further constrain the results displayed in Fig. S12. These additional constraints were:

- The pMN-p3 boundary width to be at least twice the size of the WT as explained above.
- The pMN-p3 boundary position to be between [0.17 0.25] (as the WT boundary position is at 0.17 and some of the *in vivo* mutants show a small dorsal shift).
- The p2-pMN position to be below or equal to 0.5 (WT boundary is at 0.55, this means a reduction of the domain size of at least 15% with respect to WT) but higher than the pMN-p3 boundary position, such that the pMN domain does not disappear.
- Other aspects of patterning not to be disturbed.

The resulting retained networks present a substantially reduced parameter space and are shown in Fig. S13. From these parameter sets we took a representative point as our model for the O2e33$^{-/-}$ mutant; as expected this replicates the observed experimental phenotypes. Furthermore, we randomly tested three other resulting parameter sets and found a similar dynamical behaviour to that of our representative point.

Development • Supplementary information

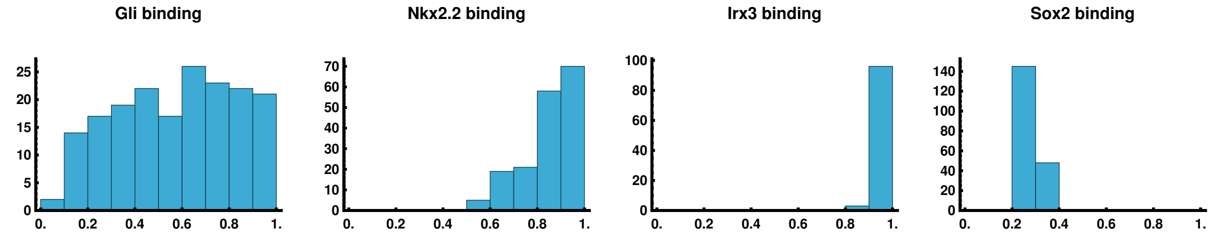

Figure S13: **Distribution of parameter changes to mimic *in vivo* O2e33$^{-/-}$ mutant.** Equivalent histograms to Fig. S12 with the additional constraints from *in vivo* observations: ventral shift of pMN-p2 boundary *and* broad p3-pMN boundary. The main results are: maintaining WT levels of Irx3 input; substantial reduction in Sox2 input, some reduction in Gli input but with a broad distribution, and a mild reduction in Nkx2.2 input.

Development • Supplementary information

F Screening three-node networks for precision

Defining a functional form

To perform a parameter screen we explored three-node networks with all possible interactions between the nodes, as this has provided useful insights in other systems (Fig. 1A) [Cotterell and Sharpe, 2010, Leon et al., 2016]. For the purpose of exploring different dynamics, we enumerated the different possible transcriptional/occupancy states of the promoter to model the production rates of a given protein. These rates depend on polymerase availability, signal input (morphogen) and regulating transcription factors, with concentrations x_{p}, x_{in} and x_i respectively. The transcription factors i can be activating ($i \in \mathcal{P}$) or repressing ($i \in \mathcal{N}$), with $\mathcal{P}$ and $\mathcal{N}$ denoting the sets of activating and repressing transcription factors, respectively. While in the previous model, in its most general form (C.1a), different protein production rates can be used for different DNA conformations, in the neural tube network we used the same production rate for all protein-producing input conformations (see Supp. E). We adopt the same approach here and set the production rate to unity in appropriate units of time; thus the model is specified only by the binding affinities of the various DNA conformations. Without loss of generality we fixed the affinity (and hence the weight) of the unbound conformation to 1 as explained in [Sherman and Cohen, 2012]. We assign the weights of conformations with only one bound molecule as $k_{\mathrm{p}}x_{\mathrm{p}}$, $k_{\mathrm{in}}x_{\mathrm{in}}$ and $k_i x_i$. In accordance with our previous model (C.1a), we set the following constraints:

- All conformations with polymerase and without any repressor $i \in \mathcal{N}$ produce protein; it does not matter whether signal or any activator $i \in \mathcal{P}$ are bound.
- Conformations that have one or more repressor $i \in \mathcal{N}$ bound together with either signal, polymerase or any activator $\mathcal{P}$ are excluded, based on the assumption that these molecules compete for the same binding site
- Binding of signal or any activator $\mathcal{P}$ enhances binding of polymerase
- No other cooperativity effects are present

Expressions for conformation states

The only states that can produce protein are those with polymerase bound. For brevity we follow the convention in Supp. E and abbreviate

$$w_{\mathrm{p}} = k_{\mathrm{p}}x_{\mathrm{p}} \tag{F.1}$$

Development • Supplementary information

in the following, taking polymerase levels as constant throughout our dynamics. As specified above, the only states that can bind polymerase are those that have no repressors bound. We assume no cooperativity between signal x_{in} and activators $x_i, i \in \mathcal{P}$, hence the total weight of states that can potentially bind polymerase (assuming two binding sites per activator $i \in \mathcal{P}$ but only one for the signal) is:

$$(1 + k_{\text{in}}x_{\text{in}}) \prod_{i\in\mathcal{P}} (1 + k_i x_i)^2 \tag{F.2}$$

Given that repressors $\mathcal{N}$ can only bind by themselves, and that there is no other cooperativity between the inputs, the total weight for conformations with at least one repressor $\mathcal{N}$ bound while assuming two binding sites per repressor $i \in \mathcal{N}$ is:

$$-1 + \prod_{i\in\mathcal{N}} (1 + k_i x_i)^2 \tag{F.3}$$

In accordance with biological intuition, polymerase is recruited by activators $\mathcal{P}$ or signal. The simplest way to implement this is to increase the weight of conformations having both polymerase and at least one activator $i \in \mathcal{P}$ or signal by a cooperativity factor c, giving a total weight of:

$$c\, w_{\text{p}}[-1 + (1 + k_{\text{in}}x_{\text{in}}) \prod_{i\in\mathcal{P}} (1 + k_i x_i)^2] \tag{F.4}$$

Finally, the weight for the unbound (empty) conformation is taken as 1, as explained above, and for the conformation with one polymerase bound it is w_{p} as defined in (F.1). The total weight, i.e. the denominator of the protein production rate, is then

$$w_{\text{p}} + c\, w_{\text{p}}[-1 + (1 + k_{\text{in}}x_{\text{in}}) \prod_{i\in\mathcal{P}} (1 + k_i x_i)^2] + (1 + k_{\text{in}}x_{\text{in}}) \prod_{i\in\mathcal{P}} (1 + k_i x_i)^2 - 1 + \prod_{i\in\mathcal{N}} (1 + k_i x_i)^2 \tag{F.5}$$

while the numerator is the total weight of conformations *with* polymerase, either on its own (F.1) or together with activators or signal (F.4), giving overall for the production rate (which with protein production set to unity is also the probability of being in a DNA conformation that produces protein)

$$\frac{w_{\text{p}} + c\, w_{\text{p}}[-1 + (1 + k_{\text{in}}x_{\text{in}})\phi]}{w_{\text{p}} + c\, w_{\text{p}}[-1 + (1 + k_{\text{in}}x_{\text{in}})\phi] + (1 + k_{\text{in}}x_{\text{in}})\phi + \psi - 1} \tag{F.6}$$

with the abbreviations

$$\phi = \prod_{i\in\mathcal{P}} (1 + k_i x_i)^2, \qquad = \prod_{i\in\mathcal{N}} (1 + k_i x_i)^2 \tag{F.7}$$

Development • Supplementary information

General strong cooperativity limit

It will be convenient in the following to write the effective affinities of signal and activating TFs in combination with polymerase in a form that includes the cooperativity effect from the factor c, i.e. in terms of $\tilde{k}_{\text{in}} = c\,k_{\text{in}}$ and $\tilde{k}_i = c\,k_i$ for $i \in \mathcal{P}$. The protein production rate is then expressed as

$$\frac{w_{\text{p}} + c\,w_{\text{p}}[-1 + (1 + \tilde{k}_{\text{in}} x_{\text{in}}/c)\phi]}{w_{\text{p}} + c\,w_{\text{p}}[-1 + (1 + \tilde{k}_{\text{in}} x_{\text{in}}/c)\phi] + (1 + \tilde{k}_{\text{in}} x_{\text{in}}/c)\phi + \psi - 1} \tag{F.8}$$

with now

$$\phi = \prod_{i \in \mathcal{P}} (1 + \tilde{k}_i x_i/c)^2 \tag{F.9}$$

We can now compare with the analogous expression (E.1) in the neural tube network. There all interactions are repressive so that $\mathcal{P}$ is the empty set and hence $\phi = 1$, which simplifies (F.8) to

$$\frac{w_{\text{p}}(1 + \tilde{k}_{\text{in}} x_{\text{in}})}{w_{\text{p}}(1 + \tilde{k}_{\text{in}} x_{\text{in}}) + \tilde{k}_{\text{in}} x_{\text{in}}/c +} \tag{F.10}$$

This agrees with (E.1) except for the middle term in the denominator, which represents the weight of DNA conformations with only signal but no polymerase bound. Its absence in the neural tube network formally corresponds to the strong cooperativity limit $c \to \infty$. In our screen we use a finite cooperativity $c = 100$ to avoid the extreme case of excluding conformations with only signal bound completely; this value of c is still large enough, however, to replicate the dynamics of the neural tube network. We thus take (F.8) with $c = 100$ as the form of protein production rates in our screen; compared to the neural tube case this allows us to include both activating and repressive interactions.

Adding a protein decay term (with unit decay rate) and stochastic fluctuations, the dynamics of the three-node networks in our screen, with protein levels x_1, x_2 and x_3, is thus described by

$$\frac{d}{dt}x_j = \frac{w_{j,\text{p}} + c\,w_{j,\text{p}}\,[-1 + (1 + k_{j,\text{in}} x_{\text{in}}/c)\phi_j]}{w_{j,\text{p}} + c\,w_{j,\text{p}}\,[-1 + (1 + k_{j,\text{in}} x_{\text{in}}/c)\phi_j] + (1 + k_{j,\text{in}} x_{\text{in}}/c)\phi_j + {}_j - 1} - x_j \tag{F.11}$$

$$\phi_j = \prod_{i \neq j} (1 + [k_{ji}]_+ x_i/c)^2$$

$${}_j = \prod_{i \neq j} (1 + [k_{ji}]_- x_i)^2$$

for $j = 1, 2, 3$; compared to (F.8) we have dropped all tildes to unclutter the notation. We have also allowed the sets $\mathcal{P}$ and $\mathcal{N}$ of activating and repressing transcription factors to be determined implicitly by the system parameters. This is done by generalizing the affinities k_{ji}

Development • Supplementary information

so that a positive sign indicates an activation of j by i and a negative sign a repression. The corresponding switching of species i between the products over activators and repressors is achieved mathematically by setting $[k]_+ = \max(k, 0)$ and $[k]_- = \max(-k, 0)$.

To mimic the structure of the neural tube network, we assume that only proteins 1 and 2 have direct signal inputs, while 3 does not, so that $k_{3,\text{in}} = 0$. This leaves 11 network parameters: 2 for the signal (gradient) inputs from the gradient ($k_{1,\text{in}}$ into node 1 and $k_{2,\text{in}}$ into node 2), 6 from the interactions between TFs (k_{12}, k_{13}, k_{21}, k_{23}, k_{31} and k_{32}) and 3 for polymerase binding weights ($w_{1,\text{p}}$, $w_{2,\text{p}}$ and $w_{3,\text{p}}$).

Parameter exploration

We explored the 11 dimensional parameter space specified above using a uniform log distribution ($\log_{10}$), where the ranges are set differently depending on the parameter. Specifically we chose the ranges as: $\text{range}(k_{\text{in}}) = [10 : 400]$, $\text{range}(w_{\text{p}}) = [0.1 : 10]$, $\text{range}(k_{ji}) = [-100 : -1] \cup [1 : 100]$ with the sign of each regulation k_{ji} being chosen randomly.

We provide a schematic in Fig. S14 of the sequential steps taken to screen for relevant networks, analyse them and classify them into topologies. We explored parameter combinations for a three-node network defined in the form (F.11). The main criterion for choosing a viable set of parameters was that they must produce a patterned steady state, i.e. a saddle-node bifurcation on the same gradient as in the neural tube, defined as $x_{\text{in}} = e^{-s/0.15}$ where s indicates dorsal-ventral neural tube position and ranges from 0 to 1. To avoid trivial effects from shifts in the boundary position we set a further constraint that the bifurcation must occur at a position s in the same range as in the neural tube network, $0.165 \leq s \leq 0.17$. More specifically networks were required to be monostable below $s = 0.165$, with high levels of x_1; and bistable beyond $s = 0.17$, with one state having high x_2 and the other high x_1 (with "high" being a concentration value above 0.6). For each network meeting these criteria, we then proceeded to calculate the MAPs in the same way as for the neural tube network (as explained in Supp. C), and the jump time. We selected networks that have boundaries sharper than a certain threshold, set by requiring the boundary to be no wider than 0.2 fractional neural tube units; boundary widths were calculated based on their transition time obtained from simulating the SDEs. To simulate the neural tube network from (E.1) in the screen we used the standard parameters from that network, reverting to the original version [Cohen et al., 2014] with maximal concentrations of unity for all TFs in order to ensure comparability with the networks produced by the screen. We removed all terms relating to Irx3, as these do not contribute substantially to the dynamics of transitioning from a pMN to a p3 steady

Development • Supplementary information

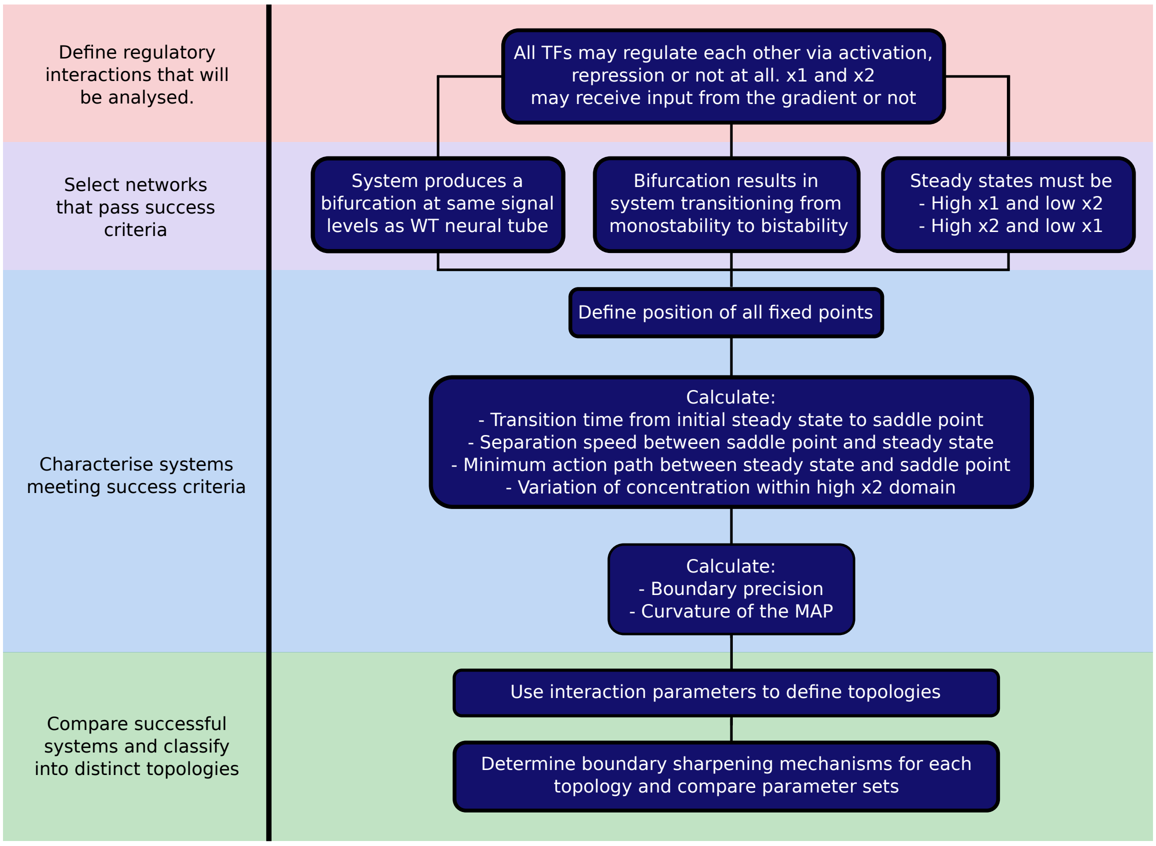

Figure S14: **Schematic of steps for systematic screening**. We desgined the screen to first identify parameter sets that describe networks that generate a sharp boundary at a specific location within a gradient. This ensures that the resulting networks are comparable with each other. The parameter sets that pass this filter are then analysed by defining the characteristics relevant to forming a precise boundary. Finally, we classify the parameter sets into topologies.

state. We further set production and degradation rates to be equal to unity in the screen as these simply scale the jump time and do not affect the results.

In analysing the results of the network screen we quantified the curvature of the MAP as the largest perpendicular distance of any point on the MAP from the straight line between steady state and transition point, normalised by the total length of this line. We refer to this value throughout the text by the shorthand "curvature" as it gives a quantitative indication of how much the MAP deviates from the shortest path. This metric was normalised by the saturation values of each species to control for possible distortions due to differing typical concentration levels of different molecular species. Additionally we also tried a Euclidean metric in logarithmic concentrations and obtained similar results.. The curvature was measured at $s = 0.25$ and the robustness of the results with respect to this choice of neural tube position was tested by comparing with multiple other locations, with qualitatively similar results in all cases (data not shown).

Development • Supplementary information

In the analysis we also characterised networks by the strength of the contribution of the third node, which does not receive direct signal input. We quantified this by taking the value of x_3 at the steady state and transition point (saddle point) and multiplying each by parameters for the repression or activation of nodes 1 and 2 by node 3, taking the maximum value. The multiplication by representative concentration levels of the third node was motivated by the fact that when those concentrations are small, even large interaction parameter values have small net effects.

Networks with a low third node contribution are effectively two-node networks, and turned out to have low MAP curvature. This led us to explore other mechanisms for generating sharp boundaries. Geometrically, in the space of expression levels (phase space), the speed at which the steady state and saddle point separate as a function of neural tube position s is a plausible contributor to boundary sharpness because even if the fluctuations around the initial steady state favour a jump, such a jump will be inhibited by a large separation between steady state and transition point. High signal sensitivity should thus lead to rapidly increasing jump times and hence to sharp boundaries. To measure signal sensitivity we focussed on a fixed position (chosen as $s = 0.25$) along the neural tube, beyond the saddle-node bifurcation, and calculated the Euclidean distance between steady state and transition point. We then used this as a simple quantitative indication of signal sensitivity. We checked the robustness of this measure by performing the measurement for different *fixed* positions along the neural tube, and also at *variable* locations chosen as the centre of the boundary region for each network; we found qualitatively similar results in every case (data not shown).

When a network had a high signal sensitivity, this typically resulted in the steady state (the expression profile) of x_2 varying, i.e. changing within a domain of the steady state pattern. We quantified this heterogeneity by the standard deviation of x_2 within the region of high x_2 expression. This confirmed (see Fig. S15) that sharp 2D networks have a higher level of heterogeneity than 3D networks, which use the curvature of the MAP to generate sharpness.

Characterisation of topologies

Finally we analysed the topologies of the networks resulting from the screen. To sort networks into topologies we used thresholds to identify whether nodes 1 and 2 receive significant signal input, and for each of the TF nodes whether it significantly activates or represses the other TFs. Starting with the former, within the input parameter range $[10 : 400]$ for nodes 1 and 2, we took any parameter $30 < k_{\text{in}}$ to be a positive input; lower values were classified as lack of input. This cutoff was chosen by testing a range of different values and imposing the

Development • Supplementary information

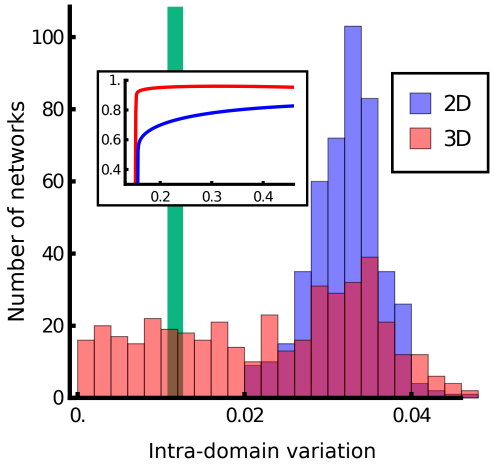

Figure S15: Histogram of variation of the expression level of the second node within its domain of expression for 3D (red) and 2D (blue) networks; inset shows example variation of expression levels across a domain. 3D networks can generate domains of expression with more constant levels of expression (lower domain variation) than 2D networks, which rely on signal sensitivity to create sharp boundaries. Green line represents the WT network.

constraints that we want to neither classify the majority of networks as having two inputs (which would provide no information on the input topology, as could happen if the cutoff was too low) nor assign any network to a topology with no inputs (which would not make biological sense and would occur when the cutoff is too high). For interactions between nodes we took into account not only the parameters k_{ji} but whether each parameter in conjunction with the actual states of the system would have a noticeable effect. We evaluated interactions by considering the contribution of an interaction given the highest level that the effector node can take. Accordingly, we consider an interaction with $0.3 < |k_{ji}| \max(x_i)$ to be significant, otherwise we classify it as negligible. The maximum was taken over all steady states for all neural tube positions. The cutoff value of 0.3 was chosen by systematic inspection of a representative number of networks, for which we compared the dynamics with and without individual interactions and assessed whether these were qualitatively identical or not. To assess the robustness of the cutoff value, we varied it within a range up to an order of magnitude larger and found that the results of our characterisation of network topologies remained qualitatively the same (data not shown).

With this approach we classified all the 3D network parameter sets into topologies, determined those that occurred most often (Fig. S16) and plotted the boundary precisions they generate (Fig. 5H). The results indicated that although some topologies are more frequently represented amongst networks producing a sharp boundary, there is no single topology that ensures sharpness. Some networks (such as 1–4 in Fig. S16) prevented the boundary from

Development • Supplementary information

becoming very imprecise, but even within these network topologies the range of sharpness was large (Fig. 5G,H & Fig. S16 & Fig. S17). This leads to the conclusion that the dynamical properties generated by the network, rather than the structure of the network determines boundary precision. Indeed, we confirmed by analysing each topology separately that the main indicators of sharpness are the two mechanisms identified in the main text: curvature of transition path and signal sensitivity (Fig. S17). Nonetheless, a network's topology can substantially bias the dynamics towards high MAP curvature, and hence towards sharpness.

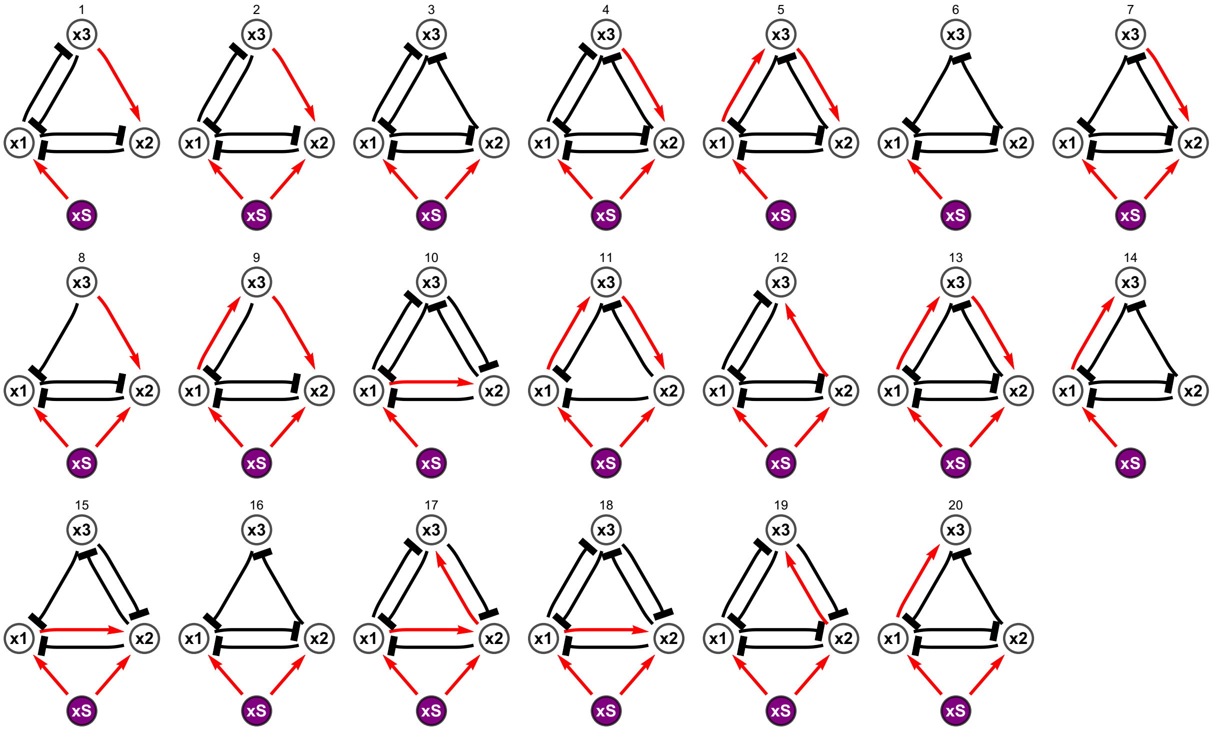

Figure S16: List of topologies that generate sharp boundaries, sorted in the same order as Fig. 5**H**. Red arrows indicate activation, black lines with blunt ends represent repression. Mutual repression between the first and second nodes (1 and 2) is a consistent feature, as well as the input from the signal to the first node. For the sharpest networks, a mutual repression between the first and third nodes is observed.

Development • Supplementary information

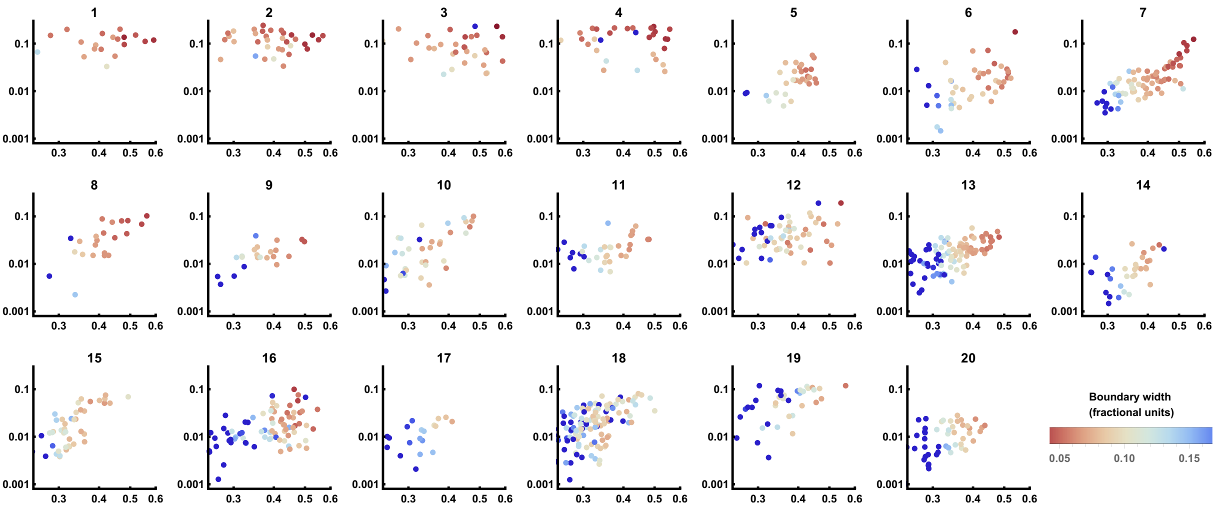

Figure S17: MAP curvature plotted against signal sensitivity with boundary width indicated by colouring. The data are equivalent to those shown in Fig. 5**D**, but here each plot represents an individual network topology and networks with wide boundaries have been included in the plots (deep blue). Network topologiess are ordered as in Fig. S16. While signal sensitivity does not exhibit obvious differences between topologies, network topologies 1–4 have consistent high curvature.

Effect of signalling noise on boundary precision

We explored what effect noise in the signal gradient would have on the precision of boundaries generated by the mechanisms revealed in the screen. To this end, we simulated networks recovered from the screen using a noisy signal as an input. For this we have used Ornstein-Uhlenbeck noise and explored systematically a range of fluctuation timescales and noise amplitudes (see Eq. F.12). As is commonly done we use a log version to avoid negative values, i.e. we write the fluctuating signal input as $s_{\mathrm{OU}}(t) = \exp(\ell(t))$ where $\ell(t)$ evolves in time as

$$d\ell(t) = \theta\left(\ln(s) - \ell(t)\right) dt + \sigma dW(t) \tag{F.12}$$

$$\ell(0) = \ln(s) \tag{F.13}$$

The variables are the standard terms for Ornstein-Uhlenbeck processes: θ is the inverse correlation time of fluctuations, σ is the noise amplitude, W is a Wiener process, s is the constant Gli input in the original model. We compared the boundary widths generated by simulations using these noisy gradients with those in which the signal was constant, for otherwise identical parameter sets (Fig. S18). This revealed that noise in the signal had relatively limited effects on the precision of boundaries for moderate levels of noise. Moreover, the same relative sharpness of boundaries for the different networks was found in the simulations with a constant and a noisy signal. Above a level of signal noise all sharpness was lost, as anticipated. Thus the

Development • Supplementary information

determining factors for boundary sharpness are curvature and signal sensitivity, as the networks that maximise these two parameters produce the sharpest boundaries with or without signal noise (Fig. S18).

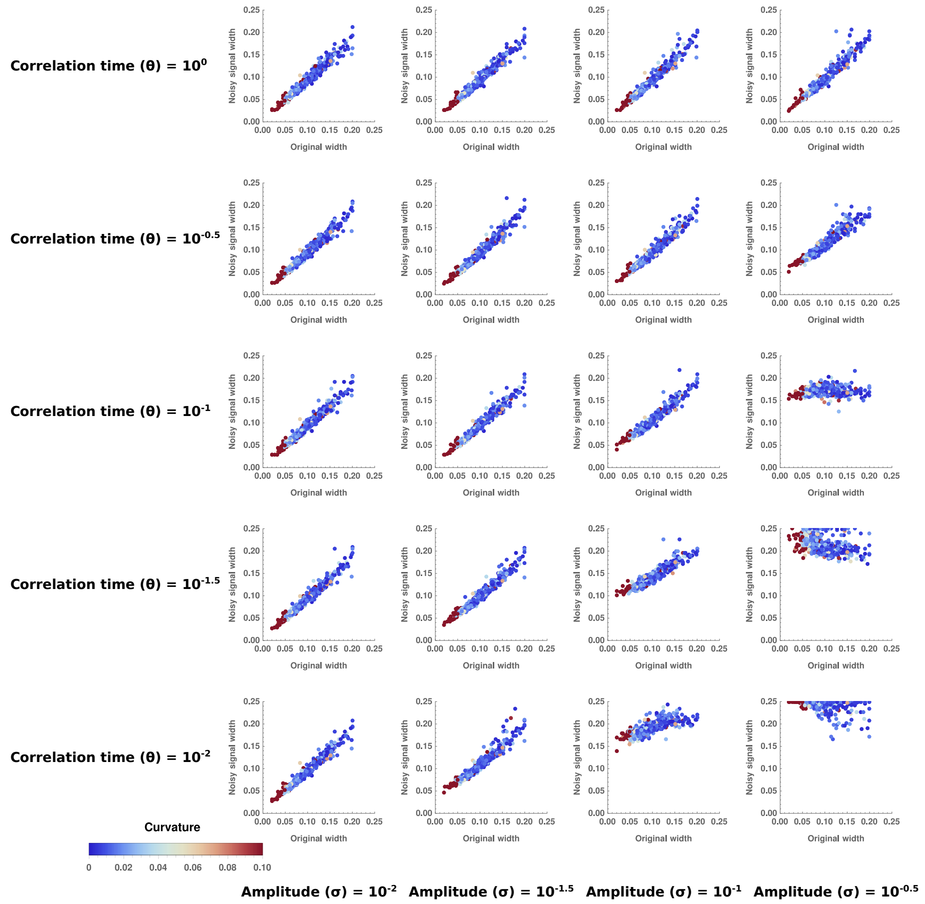

Figure S18: Effect of different levels of noise in the gradient on boundary precision. The boundary widths produced by systems recovered from the computational screen are plotted for simulations with no noise in the signal gradient (x axis) and with noise in the signal gradient (y axis). Colour labels networks from least (blue) to most (red) curvature. The behaviour of the noise in the signal has been modelled as an Ornstein-Uhlenbeck process, with the indicated amplitudes and correlation times. The same network parameter values were used for the simulations with and without signal noise. The analysis shows that the noise in the signal has relatively small effects on the precision of boundaries, except when the noise in the signal is so extreme that all sharpness is lost (bottom right plots).

Comparison with Drosophila GAP gene and Eye Imaginal disc networks.

We compared the networks recovered from the computational screen with those described for anterior posterior patterning of the Drosophila embryo and eye imaginal disc [Verd et al., 2017, Graham et al., 2010]. Both these systems have been characterised extensively such that

Development • Supplementary information

we have sufficient knowledge of the network to perform our analysis [Akam, 1987, Ingham, 1988, Sánchez and Thieffry, 2001, Manu et al., 2009, O'Neill et al., 1994, Rebay and Rubin, 1995]. We added intrinsic noise to the original models from [Verd et al., 2017, Graham et al., 2010] using Langevin equations and an Ω that was chosen to result in fluctuations without leading to ergodicity. For the GAP gene system we used the parameters and equations as described in [Verd et al., 2017], for the imaginal disk network we used the Mathematica code provided as supplementary in [Graham et al., 2010]. We inspected the configurations in gene expression fluctuations near relevant steady states.

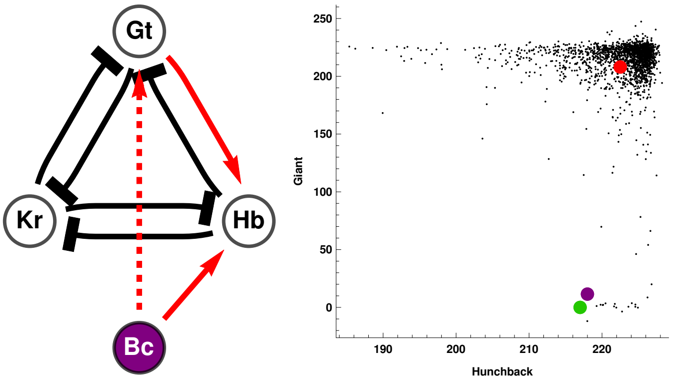

Figure S19: (Left) GAP gene network for the anterior boundary between Kruppel and Giant. The network has the same topology as the most frequently recovered network identified by our screen, with a difference only in the input (dashed link). This differences does not affect the dynamics as it only alters how the network interprets a change in signal. (Right) Black dots represent multiple simulations and illustrate fluctuations near the high-Giant / low-Kruppel steady state (red point). To transition to the low-Giant and high-Kruppel steady state (green point) the system must reach the transition point (purple point). The fluctuations in gene expression space are coerced into the Hunchback dimension (x axis), decreasing the probability of a stochastic fluctuation of the system reaching the transition point.

The architecture of the transcription circuits that comprise the GAP gene network [Verd et al., 2017] match closely those found in our computational screen (Fig. S16). As predicted by our computational screen, we can identify role for specific links between network components in the formation of GAP gene boundaries. In particular, for the anterior boundary between Giant and Kruppel, if we remove Knirps, which is not expressed in either of these domains, we find one the most common topologies recovered from our screen, with the correspondence Kruppel – x_1, Hunchback – x_2, Giant – x_3 (Fig. S19). In this case, Hunchback and Giant display mutual exclusivity and the graded expression profile of Giant suggests that signal sensitivity is used to sustain the sharp boundary; this is similar to the role played by Pax6 (x_3) in the neural tube GRN. An interesting difference is that while Hunchback affects the direction of fluctuations in gene expression space, it does not change in concentration and simply alters

Development • Supplementary information

the dynamics of the transition (Fig. S19).

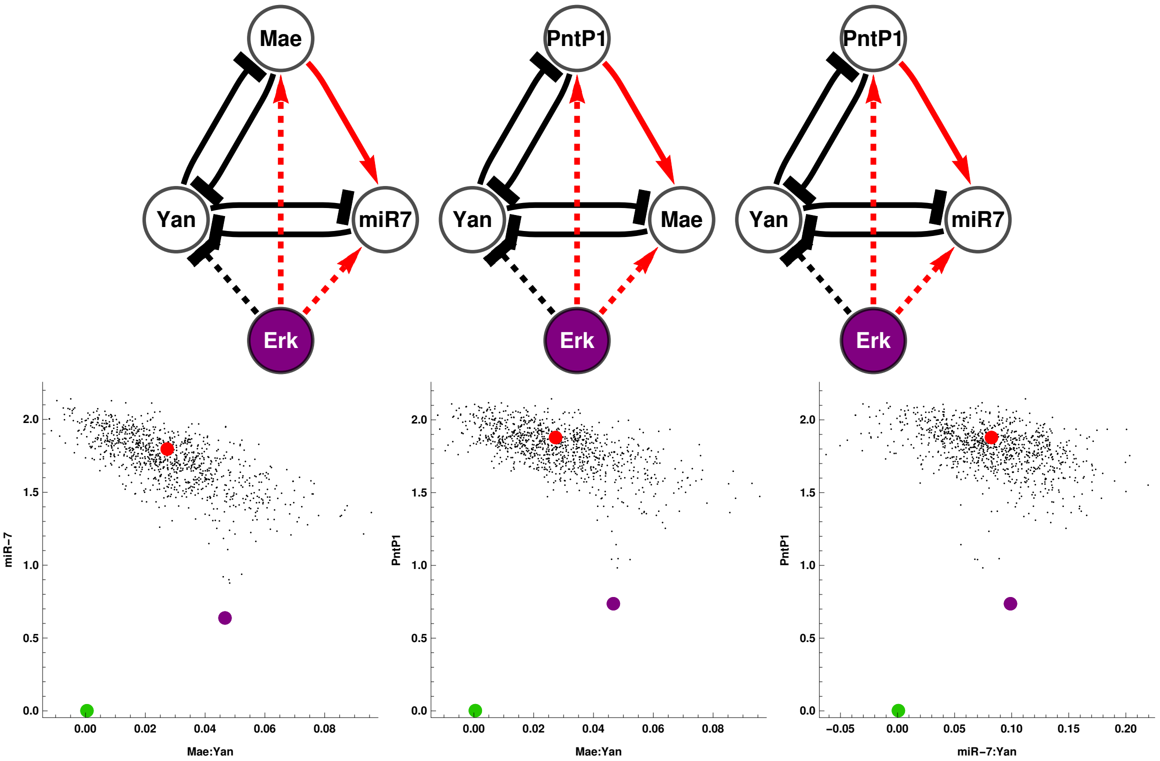

Figure S20: (Top) Multiple instances of one of the top topologies for boundary sharpness found in our computational screen are contained in the Drosophila eye disc network. This network is composed of several interactions and mediates the transition between Yan-on and Yan-off states. The configuration of inputs from the signal (Erk) is different to our topologies (dashed lines) but this does not affect the dynamics. (Bottom) Fluctuations near the Yan-off state (red point) for different projected views corresponding to the networks shown. The fluctuations are configured in directions that are not aligned with the transition point (purple point). This configuration decreases the possiblity of a cell reverting to a Yan-on state after the wave of Erk has shifted the system to a Yan-off state. Note that for Mae and miR-7 the inhibitions of Yan happen through direct interactions, thus where noted we show the fluctuations for the variable tracking the Inhibitor:Yan complex (Mae:Yan or miR-7:Yan).

The differentiation pattern of the eye imaginal disc also relies on cross-repressive interactions [O'Neill et al., 1994, Rebay and Rubin, 1995, Graham et al., 2010]. The expression of Yan, downstream of RTK signalling distinguishes between differentiated and undifferentiated precursors in the eye disc as the furrow migrates. We inspected the network proposed to achieve this [Graham et al., 2010] by focusing on three-node networks that involved Yan and two other components in cross-repression with Yan. This approach resulted in three versions of a network topology found frequently as one with high curvature in our screen, with the mappings: (1) Yan – x_1, miR-7 – x_2, Mae - x_3, (2) Yan – x_1, Mae – x_2, PntP1 – x_3 and (3) Yan – x_1, miR-7 – x_2, PntP1 – x_3 (Fig. S20). Simulations also indicate that the dy-

Development • Supplementary information

namics of these networks configure gene expression fluctations to decrease the probablity of a noise driven transition (Fig. S20). The bistable network facilitates a sharp switch between steady states, ensuring that cells only transition from a Yan-off to a Yan-on state when Yan is sufficiently activated by Erk signalling. Once the wave of Erk has passed, the dynamical curvature established by the network ensures that cells do not transition back to a Yan-on state (Fig. S20). Thus both Drosophila embryo and eye imaginal disc networks appear to have adopted network structures that are compatible with precision enhancing mechanisms.

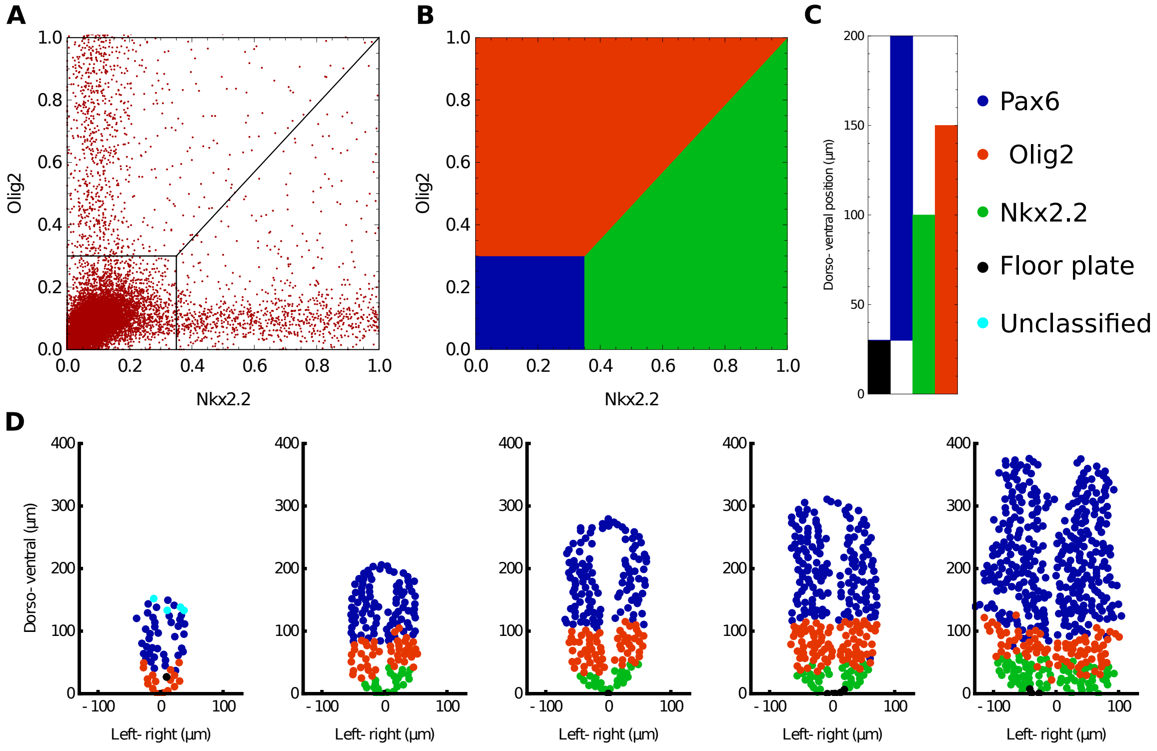

Figure S21: **Analysis of gene expression in embryos**(**A**) Plot illustrating the concentrations of Nkx2.2 and Olig2 for all cells analysed. This highlights that the majority of cells are negative for both TFs and also that very few cells co-express both TFs. (**B**) Criteria to determine the identity of each cell by using the levels of Nkx2.2 and Olig2; colours indicating cell assignment as Olig2 (red), Nkx2.2 (green) and neither (blue) are consistent throughout the figure. The concentration of Pax6 is not used for classification. (**C**) Positional limits along the neural tube for each cell type. Cells that express neither Olig2 nor Nkx2.2 are classified based on their position as they can be ventral floor plate cells (black) or more dorsal progenitors. Cells that have mismatching values of concentration and position are classified as exceptions in Cyan (**D**) Examples of classified embryos of increasing age, illustrating the accuracy of the approach for determining cell type.

Development • Supplementary information

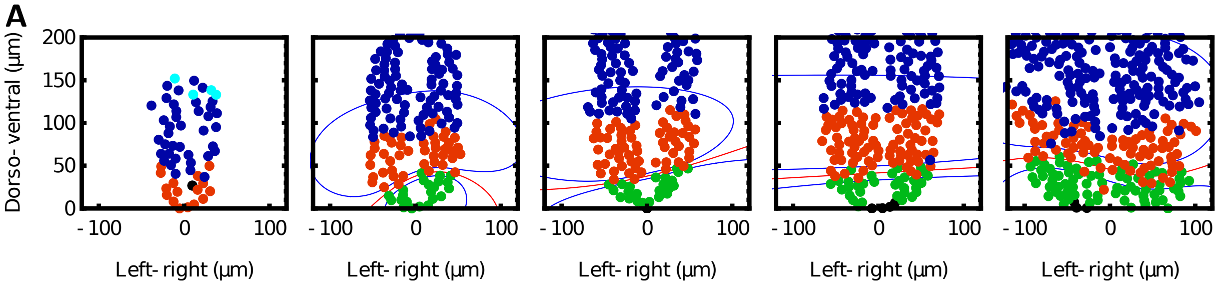

Figure S22: **Examples of boundaries determined by the Gaussian process classifier**. The red lines indicate the computed boundary position, and correspond to the image locations where the probability of being a p3 or pMN cell is 0.5. Blue lines close to the p3-pMN boundary delimit the area identified as the boundary region, where the probability of being a p3 cell is in the range 11% to 89%. By measuring the area between the two blue curves and dividing by the width of the embryo we are able to quantify the width of the boundaries. In turn by obtaining the average position of the red line, we are able to calculate the boundary position. Note that these boundaries are trained from the data and therefore require no user input as to the position of the boundary

	WT	O2e33$^{-/-}$	Pax6$^{-/-}$
150 - 250 μm	17	5	5
250 - 350 μm	13	13	3
350 - μm	16	11	8

Table S1.

Development • Supplementary information
